# Paracrine activin B-NF-κB signaling shapes an inflammatory tumor microenvironment in gastric cancer via fibroblast reprogramming

**DOI:** 10.1186/s13046-023-02861-4

**Published:** 2023-10-19

**Authors:** Yangbing Jin, Qu Cai, Lingquan Wang, Jun Ji, Ying Sun, Jinling Jiang, Chao Wang, Junwei Wu, Benyan Zhang, Liqin Zhao, Feng Qi, Beiqin Yu, Jun Zhang

**Affiliations:** 1 Department of Oncology, Ruijin Hospital, Shanghai Jiao Tong University School of Medicine, No. 197 Ruijin er Road, 200025 Shanghai, China; 2 Department of General Surgery, Ruijin Hospital, Shanghai Jiao Tong University School of Medicine, No. 197 Ruijin er Road, 200025 Shanghai, China; 3 Department of General Surgery, Shanghai Institute of Digestive Surgery, Ruijin Hospital, Shanghai Jiao Tong University School of Medicine, No. 197 Ruijin er Road, 200025 Shanghai, China; 4 Department of Pathology, Ruijin Hospital, Shanghai Jiao Tong University School of Medicine, No. 197 Ruijin er Road, 200025 Shanghai, China

**Keywords:** Gastric cancer, Activin B, Fibroblast reprogramming, NF-κB, Tumor microenvironment

## Abstract

**Background:**

Important roles of INHBB in various malignancies are increasingly identified. The underlying mechanisms in gastric cancer (GC) microenvironment are still greatly unexplored.

**Methods:**

The clinical significance of INHBB and the correlation between INHBB and p-p65 in GC were assessed through analyzing publicly available databases and human paraffin embedded GC tissues. The biological crosstalk of INHBB between GC cells and fibroblasts was explored both in vitro and in vivo. RNA-seq analyses were performed to determine the mechanisms which regulating fibroblasts reprogramming. Luciferase reporter assay and chromatin immunoprecipitation (CHIP) assay were used to verify the binding relationship of p65 and INHBB in GC cells.

**Results:**

Our study showed that INHBB level was significantly higher in GC, and that increased INHBB was associated with poor survival. INHBB positively regulates the proliferation, migration, and invasion of GC cells in vitro. Also, activin B promotes the occurrence of GC by reprogramming fibroblasts into cancer-associated fibroblasts (CAFs). The high expression of INHBB in GC cells activates the NF-κB pathway of normal gastric fibroblasts by secreting activin B, and promotes fibroblasts proliferation, migration, and invasion. In addition, activin B activates NF-κB pathway by controlling TRAF6 autoubiquitination to induce TAK1 phosphorylation in fibroblasts. Fibroblasts activated by activin B can induce the activation of p65 phosphorylation of GC cells by releasing pro-inflammatory factors IL-1β. p65 can directly bind to the INHBB promoter and increase the INHBB transcription of GC cells, thus establishing a positive regulatory feedback loop to promote the progression of GC.

**Conclusions:**

GC cells p65/INHBB/activin B and fibroblasts p65/IL-1β signal loop led to the formation of a whole tumor-promoting inflammatory microenvironment, which might be a promising therapeutic target for GC.

**Supplementary Information:**

The online version contains supplementary material available at 10.1186/s13046-023-02861-4.

## Highlights

Tumor-secreted activin B promotes gastric cancer via fibroblast reprogramming.

Activin B activates the NF-κB pathway by inducing auto-ubiquitination of TRAF6 in fibroblasts.

Activin B stimulates the production of a pro-tumorigenic secretome and matrisome.

Activin B activates NF-κB pathway of fibroblasts to up-regulate IL-1β secretion.

IL-1β activates transcription factor p65 of gastric cancer to up-regulate INHBB expression.

**Supplementary Information:**

The online version contains supplementary material available at 10.1186/s13046-023-02861-4.

## Introduction

Globally, gastric cancer (GC) ranks fifth among cancers in terms of diagnostic prevalence and third in terms of major causes of death [1, 2]. Exploring the important genes that affect cell survival in the genome regulatory network is crucial for transforming molecular characterization to useful clinical application. Because tumor tissue cells and their complex relationship with microenvironment affect all aspects of tumor generation and development, the role played by genes in regulating the phenotype and function of microenvironment is worth in-depth study [3, 4].

Activin B, a member of the TGF-β superfamily, is a dimer structure formed by the carboxyl terminal domain of the inhibin β subunit (INHBB) linked by a single disulfide bond [5]. Activin pathway is a well-studied pathway that is widely expressed in all stages of body growth, where it is recognized for its many essential roles in embryonic development, disease progression, and tissue homeostasis. Through series studies, Zhang et al. uncovered that activin B/Rho A/mDia1/Cdc42 axis plays a key function in bone marrow-derived mesenchymal stromal cells (BMSCs) migration by promoting membrane ruffling, microtubule morphology, and adhesion signaling dynamics. Inactivation of activin B-Cdc42 inhibits stimulation of Golgi polarization and adipose-derived mesenchymal stem cells-mediated skin wound healing [6–8]. Furthermore, overexpressing of activin B was identified in three different models of kidney fibrosis, as well as in human kidneys with fibrosis. Sox9-activin B provides a prospective entry point to surmount kidney fibrosis [9, 10]. Nevertheless, there is still room for improvement in understanding its specific value in tumor research [11]. Recently, INHBB has been the subject of many studies concerning liver, colorectal, and prostate cancers as a new tumor promoting biomarker. For example, for liver cancer, it was found that Sox9/INHBB axis mediated crosstalk between cancer cells and hepatic stellate cells and promoted the metastasis of hepatocellular carcinoma [12]. For rectal cancer, INHBB was found to be one of indexes for predicting the efficacy of neoadjuvant radiotherapy and chemotherapy [13]. For prostate cancer, the increase of activin B was found to be related to the increase of tumor grade making it a potential prognostic biomarker of invasive prostate cancer [14].

Using data from public databases, it was found that the up-regulation of INHBB in GC is correlated with the poor prognosis of patients, where change in INHBB-related immune cell infiltration may affect the prognosis of patients with GC [15]. However, the precise role of INHBB in GC and its impact on inflammatory microenvironment remain unclear. As an extremely important part of the tumor microenvironment, cancer-associated fibroblast (CAF) is formed by normal fibroblasts residing at the edge of the tumor and will infiltrate into the tumor through functional reprogramming [16]. Thus, CAFs remodel the tumor microenvironment to influence tumor development by secreting various growth factors, cytokines, and chemokines, and reconstructing extracellular matrix (ECM) [17, 18]. Therefore, the factors that induce CAF phenotype and its molecular targets in GC still need to be fully studied. Because activin B has been proved to be pleiotropic in a variety of inflammatory diseases and fibrosis [10, 19, 20], our research purpose focus on the possible role of INHBB in GC development and its potential contribution to microenvironment crosstalk.

According to our study, we provided evidence that INHBB can promote the proliferation, migration, and invasion of tumor cells. At the same time, we identified activin B as an important participant in the differentiation of GC fibroblasts. Hence, the activated fibroblasts create a pro-tumor microenvironment, which can increase the tumor growth of GC through a positive feedback loop. Thus, we have provided a promising target in guiding the next-generation therapies of GC.

## Materials and methods

### Cell culture

The GC cell lines MGC-803, HGC-27 and MKN-45 used in our study were purchased from the Shanghai Institute of Life Sciences, Chinese Academy of Sciences. Fibroblasts were isolated from 10 independent GC patients who underwent radical gastrectomy in Ruijin Hospital, Shanghai Jiao Tong University School of Medicine. Normal fibroblasts originated from adjacent non-tumor tissues at least 5 cm away from the resection edge of tumor tissue. The tissue was divided into small pieces of about 2 mm [3] and was inoculated on a 10 cm in diameter petri dish. After 7 days of culture, a uniform fibroblast group was produced, which was then expanded into 10 cm in diameter culture dishes for subsequent passage amplification. Our experiment ensured that each fibroblast population maintained the amplification within 10 generations to minimize clonal selection and culture stress, which may occur during extended culture. All cells were cultured in Dulbecco’s Modified Eagle Medium (DMEM) containing 10% fetal bovine serum, 100 U/mL penicillin. The incubator environment was always at 5% CO_2_ at 37 °C.

### Human gastric cancer tissue immunohistochemistry staining

Human GC tissues (containing 90 gastric tumor tissues and 90 para-carcinoma tissues) was used to verify the relationship between INHBB, p-p65 and clinicopathological characteristics. All patients were diagnosed as GC by pathology. Immunohistochemistry (IHC) staining was used to analyze the protein levels of INHBB and p-p65 in gastric tumor tissues and matched adjacent normal tissues. The staining intensity of each sample was scored according to the color intensity (0: no staining; 1: weak staining; 2: moderate staining; 3: strong staining) and the range of positive staining cells (1: 0–25%; 2: 26–50%; 3: 51–75%; 4: 76–100%). The IHC score was determined by multiplying the staining intensity score by the staining cell range score. Each sample was scored independently and the results were summarized. A score of 6 or less was defined as low expression of IHC score, and a score higher than 6 was defined as high expression of IHC score. Information on antibodies used is described in detail in Supplementary Table S1.

### Immunofluorescence assay

In the immunofluorescence assay, cells were fixed with 4% paraformaldehyde for 30 min and washed with PBS for 3 times. Then, the cells were incubated with Triton for 15 min, washed with PBS for 3 times, and then sealed with serum at 37 °C for 30 min. Afterwards, BSA was discarded and incubation was carried out with the first antibody at 4 °C overnight. The cells were washed with PBS 3 times, and were incubated with fluor secondary antibody for 1 h. Afterwards, they were washed again with PBS 3 times, and were incubated with 1 µg/mL DAPI for 5 min. After sealing with ProLong™ Gold anti-quenching agent, images were taken with fluorescence microscope for positioning. Information on antibodies used is described in detail in Supplementary Table S1.

### Western blot

Under ice for 1 h, the tissues were lysed using RIPA lysis buffer containing 1mM PMSF (note: cells were lysed under the same conditions for 10 min), and centrifuged at 13,000 rpm, 4 C for 30 min. The same amount of protein was separated by 12.5% SDS-PAGE that was transferred to PVDF membrane. The protein-free fast sealing solution (Epizyme, Shanghai, China) was used for 15 min for sealing the membrane at room temperature. This was followed by incubation with the first antibody at 4 °C overnight. Washed 3 times with PBST on the next day, the membrane was incubated for 1 h at room temperature with the second antibody coupled with HRP. ECL chemiluminescence detection system (Tanon, Shanghai, China) was used for analyzing the signal, and Image J software was used to quantify the intensity of the band. GAPDH was used as the internal reference control. Information on antibodies used is described in detail in Supplementary Table S1.

### RNA extraction and qRT-PCR

With GAPDH as the internal reference gene, the CT value of each target gene in different cell lines was measured using QuanStudio 6 Flex system. The total RNA of cells was extracted by TRIzol and then reversely transcribed into 20 µL system cDNA. Afterwards, the reaction system containing 2× SYBR Green and PCR Mix for polymerase chain reaction were configured and the relative expression of cell target genes was finally calculated. Information on real-time PCR primer sequences is provided in Supplementary Table S2.

### Lentivirus transfection

For lentivirus transfection, the cells were inoculated into a 6-well plate to 60% cell density, the INHBB shRNA, INHBB overexpression lentivirus particles and their respective control particles were diluted to the appropriate concentration with OptiMEM containing 5 µg/mL polybrene, and were added to the cells for infection for 24 h. On the second day, the culture medium was replaced with DMEM containing 10% of total bovine serum for 48 h. Following transfection, the transfected cells were screened in medium containing 5 µg/mL purinomycin. Cells transfected with lentivirus particles were inoculated in a 6-well plate, and Western blot assays were used to detect INHBB protein levels. The lentivirus target sequences used were the following: shNC: TTCTCCGAACGTGTCACGT; shINHBB-1: GCCGAGTGGACGGCGACTTCC; shINHBB-2: GCCACGGTGACAGGTGGAACA; shINHBB-3: GGGACGTGCCCAACATGATTG.

### Small interfering RNA (siRNA) transfection

For siRNA transfection, siRNA was instantaneous centrifuged and dissolved in RNase free H_2_O according to the manufacturer’s instructions. Cells were transfected with Lipofectamine 2000 and OptiMEM and the final concentration of siRNA was controlled to 50 nM. The IL-1β and ACVR1C (ALK7) silence was validated by Western blot analysis in 72 h. The IL-1β siRNA sequences used were the following: NC siRNA: UUCUCCGAACGUGUCACGUdTdT, ACGUGACACGUUCGGAGAAdTdT; IL-1β siRNA-1: CCUUCAUCUUUGAAGAAGAdTdT, UCUUCUUCAAAGAUGAAGGdTdT; IL-1β siRNA-2: GAGAAGAAAGUAAUGACAAdTdT, UUGUCAUUACUUUCUUCUCdTdT; IL-1β siRNA-3: CAAUAACAAGCUGGAAUUUdTdT, AAAUUCCAGCUUGUUAUUGdTdT. The ALK7 siRNA sequences used were the following: NC siRNA: UUCUCCGAACGUGUCACGUdTdT, ACGUGACACGUUCGGAGAAdTdT; ALK7 siRNA-1: CUAUCGACAUACCUCAGAAdTdT, UUCUGAGGUAUGUCGAUAGdTdT; ALK7 siRNA-2: GCUAUUGCUCAUCGAGACAdTdT, UGUCUCGAUGAGCAAUAGCdTdT; ALK7 siRNA-3: CAUCAGUCAUGCUAACCAAdTdT, UUGGUUAGCAUGACUGAUGdTdT.

### CCK8 cell proliferation assay

The cells were inoculated into 96-well plates at the concentration of 2000/200 µL of suspension for 3–4 days. An amount of 10 µL CCK8 solution was added into the wells at the same time every day, followed by putting into the cell incubator for incubation at 37 °C for 2 h. Finally, the 96-well plate was taken out and the value of optical density (OD) was measured for each well at 450 nm wavelength with a microplate reader. Analysis and data processing were carried out, and the proliferation curve was drawn.

### Colony formation assay

The cells in logarithmic growth phase were counted, 500 of them were inoculated into 6-well plates, the plates were laid evenly fully. After 14 days of incubation, the cells were fixed with 4% paraformaldehyde and stained with crystal violet for 15 min. After washing 3 times, only colonies with more than 50 cells were counted.

### Flow cytometric assay

For cell apoptosis assay, cell supernatant and adherent cells were collected, washed 2 times with PBS, and then resuspended in 100 µL 1× Binding Buffer. Amounts of 3 µL Annexin-V-FITC and 5 µL PI were added. The cells were incubated at room temperature in dark for 15 min. An amount of 200 µL 1× Binding Buffer was added to expand the sample loading system, and the apoptotic cells were detected by flow cytometry. Each experiment was repeated 3 times.

### Transwell assay

For the in vitro detection of migration and invasion, the cells to be tested were resuspended in serum-free medium, and were inoculated in the upper chamber (8 μm aperture) with or without coating with matrix (BD Biosciences). An amount of 700 µL DMEM containing 10% FBS was added in the lower chamber. To study the effect of cytokines on the migration or invasion ability to another cells, the cells secreting cytokines were laid into the lower chamber and were cultured until attachment, and then other cells in the upper chamber were added according to the above steps. After 24 h of co-culture, the non-migrated or non-invasive cells from the upper chamber were gently removed with a cotton swab. The remaining cells were fixed with polyformaldehyde solution, stained with crystal violet, and randomly selected into three fields for counting.

### Blood and tissue collection

In this study, we included 32 histologically confirmed GC patients and 10 healthy volunteers with no history of malignant disease from our center. Following the predefined research objectives, the baseline plasma of GC patients and the plasma of healthy individuals were collected according to the manufacturer’s instructions. Blood was collected into a medical anticoagulant tube through venous puncture; afterwards, it was centrifuged within 30 min from blood collection. The plasma supernatant was stored in equal parts at −80 °C. For tissue collection, the primary lesion samples and their matched adjacent samples and normal samples from 19 patients who underwent radical resection of gastric cancer in our hospital were snap frozen in liquid nitrogen and were transferred to − 80 °C for further processing. Tissues were rinsed in ice-cold PBS to remove excess blood thoroughly and weighed before homogenization. Minced the tissues to small pieces and homogenized them in fresh lysis buffer.

### ELISA

The concentration of activin B was measured in the supernatant of GC cells transfected with different interference or overexpression lentivirus and their respective control virus or in the plasma and tissues of GC patients. All the original supernatant or plasma were diluted twice and all experiments were repeated 3 times. Activin B ELISA kit was purchased from Cloud-Clone Crop, China.

### Chromatin immunoprecipitation assay

Following manufacturer’s instructions, Chromatin Immunoprecision Kits (Cat.17–371, Millipore) were used for Chromatin Immunoprecipitation (CHIP) assays. GC cells grew to 90% confluence, and were cross-linked with 1% formaldehyde for 10 min before undergoing cell lysis on ice. The best condition was to cut the cross-linked DNA into about 200 bp by ultrasonic treatment of cell lysates for DNA fragmentation. Anti-RELA antibody or homologous IgG was used to perform immunoprecipitation. After the protein/DNA complex was eluted and inversely crosslinked with free DNA, the DNA was purified by centrifugation column, and the RELA binding site in the INHBB promoter region was detected by qRT-PCR. The primer sequence information is listed in Supplementary Table S2.

### Immunoprecipitation assay

Immunoprecipitation (IP) assays were performed using gastric normal fibroblasts. IP assays were performed by Protein A/G Agarose Bead Kit (A10022, Abmart). Briefly, 1 × 10^7^ cells were harvested in 1 mL lysis buffer, were incubated for 10 min on ice, and were ultrasonically crushed 3 times. An amount of 500 µL of cell lysate was mixed with 2 µg of anti-TRAF6 antibody at 4℃ overnight to immunoprecipitation. Followed by 3 h protein A/G agarose beads co-incubation with rotation at 4℃, these composite bonded beads were washed with washing buffer for 3 times. Antigen-antibody-agarose bead complex were resuspended using 50 µL 1×SDS sample buffer and then were separated by SDS-PAGE.

### Luciferase assay

The INHBB promoter was cloned into the luciferase reporter gene vector pGL3-Basic. Three mutant sequences of INHBB promoter were amplified by PCR and were cloned into luciferase reporter gene vector pGL3-Basic. The RELA gene sequence was constructed on pCDNA3.1 (+) vector. The primer sequences used is listed in Supplementary Table S2. According to the Genomedtech protocol, cells were co-transfected with the corresponding reporter plasmid in each experiment. TK reporter constructs were used as internal controls. The Luciferase activity was detected using the dual-luciferase report assay (Promega) system.

### RNA-seq

Primary normal fibroblasts from human stomach were stimulated with 20 ng/mL recombinant huamn activin B (rActivin B, MCE, HY-P700013AF) for 48 h, and the difference of cell transcription level was compared with that of the control group. At the same time, primary normal fibroblasts from stomach were co-cultured with HGC-27, MGC-803 and MKN-45 cells with different INHBB expression levels for 48 h, and RNA was extracted to detect the changes in the transcription level of fibroblasts. All RNA samples were tested for purity and integrity. After testing the sample proved its qualification, the sample library was constructed. The different libraries were pooled according to the effective concentration and the target offline data volume. Afterwards, sequencing was performed using Illumina PE150. Finally, bioinformatics data analysis was carried out.

### In vivo assay

Male BALB/c nude mice were purchased from PHENOTEK Animal Technology Co., Ltd. (Shanghai, China). GC cells stably transfected with shRNA or overexpression lentivirus with normal gastric fibroblasts were cultured to logarithmic growth stage and injected subcutaneously into the armpit of mice (GC cells: 5 × 10^6^/200 µL per mouse; fibroblasts: 1 × 10^6^/200 µL per mouse). Tumor volume was calculated as (a×b×b)/2 (a = maximum diameter; b = minimum diameter). Three weeks later, all mice were sacrificed and the subcutaneous tumors were weighed. All experiments were approved by the Animal Protection Committee of Ruijin Hospital, Shanghai Jiao Tong University School of Medicine.

### Statistical analysis

All results were summarized as mean ± SEM (SEM = standard error of mean) values. The experimental data were analyzed by two-tailed student t-test and one-way ANOVA. Overall survival (OS) curves were generated using the Kaplan-Meier method with log-rank test comparison. The chi-square test was used to analyze the correlations between the expression of INHBB and the clinicopathologic characteristics of GC patients. Univariate analysis of variance and multivariate analysis of variance were used to evaluate the clinicopathological characteristics of patients and their relationship with prognosis. P value less than 0.05 was considered statistically significant.

## Results

### INHBB expression was up-regulated in GC and indicated poor prognosis

**Fig. 1:**
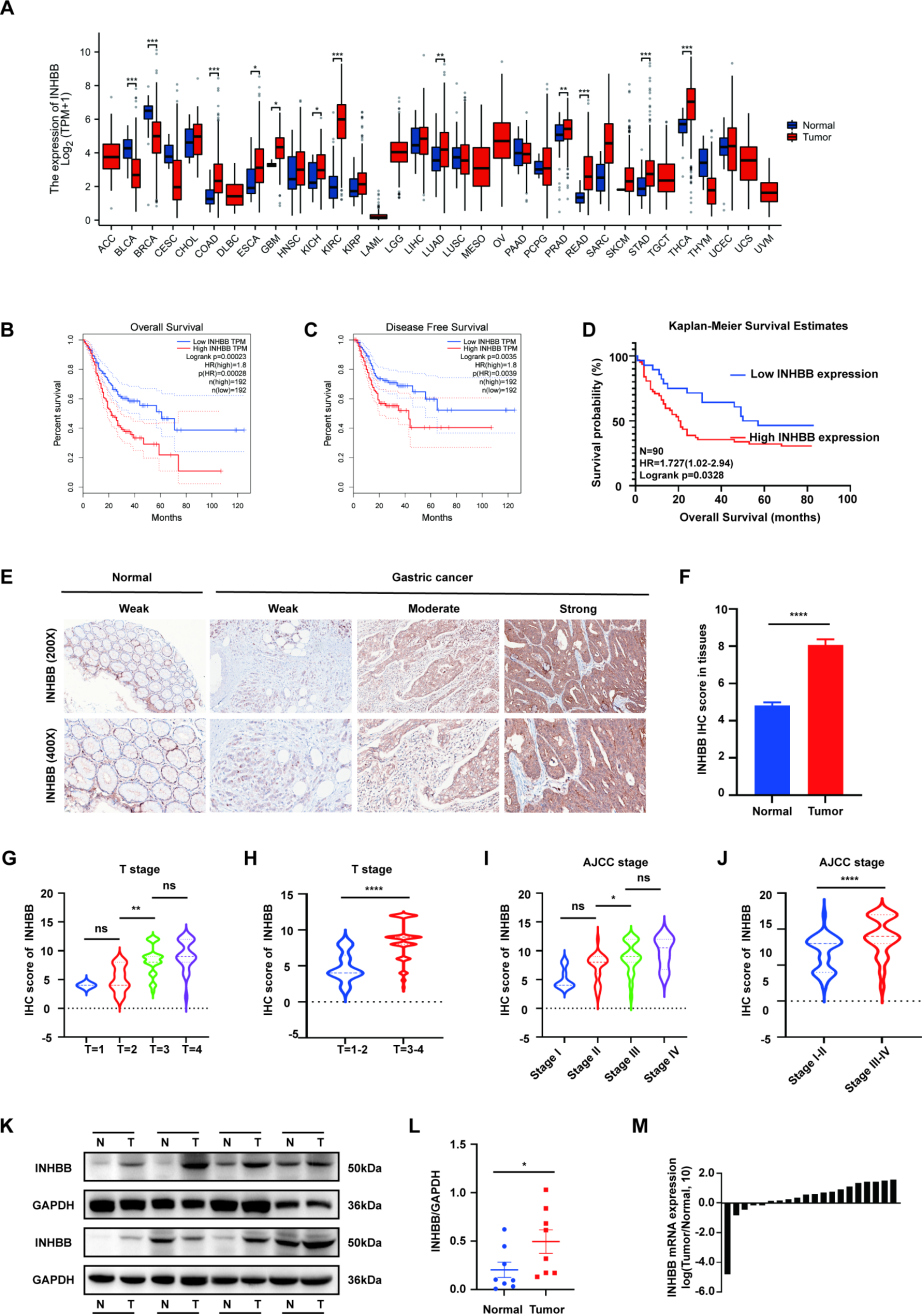
The high expression of INHBB identified in GC is correlated with poor prognosis. **A**. Up-regulation of INHBB in digestive system cancers according to TCGA database. **B-C**. Overall survival and disease-free survival analysis of the GC cohort in TCGA, based on INHBB stratified expression levels (Log-rank test). **D**. Kaplan-Meier analysis of overall survival time with GC cohort (Log-rank test) (n = 90). **E**. Representative images of INHBB IHC in GC and normal tissues with different staining intensity. **F**. Immunohistochemistry (IHC) scores of INHBB of patient samples in GC cohort (n = 90). **G-J**. Association between the IHC scores of INHBB and different T stages and AJCC stages from GC cohort (n = 90). **K-L**. Western blot analysis of the INHBB protein levels in Ruijin GC samples (n = 8). M. qRT-PCR analysis of INHBB mRNA expression in GC and matched normal tissues (n = 20) of Ruijin GC samples. *, P < 0.05; **, P < 0.01; ***, P < 0.001; ****, P < 0.0001

In order to investigate the potential roles of INHBB in cancer, INHBB pan-cancer expression levels in adjacent tumor and tumor tissues were analyzed based on The Cancer Genome Atlas (TCGA) database. We found that INHBB was up-regulated in digestive system cancers including gastric, colorectal, and esophageal cancers (Fig. 1A). In addition, using two GEO (http:/www.ncbi.nlm.nih.gov/geo/) datasets (GSE26899, GSE54129), we found an increase in INHBB levels among GC tissues compared to normal gastric samples (Fig. S1A-B). To further verify the relationship between the expression of INHBB in GC prognosis, we first used GEPIA (http://gepia.cancer-pku.cn/) TGGA survival analysis and two GEO datasets (GSE62254, GSE84437), which showed high expression levels of INHBB in tumor tissue were correlated with the worse overall survival (OS) and disease-free survival (DFS) time of GC patients (Fig. 1B-C, S1C-D). Next, we used a cohort of 90 patients with GC and their matched paracancerous samples for IHC staining. Thus, it was also confirmed that in GC patients a higher expression of INHBB was associated with a worse OS time compared to a lower INHBB expression (P = 0.0328, Log-rank test), and the expression of INHBB protein in tumor tissues was higher than that in adjacent non-tumor tissues, which was consistent with the results of TCGA analysis (Fig. 1D-F). To assess the possible role of INHBB in the progression of GC, the relationship between some important clinicopathological characteristics and INHBB expression levels was evaluated in GC cohort. The results showed that a higher INHBB staining score was related to a later T stage and a later TNM stage (Fig. 1G-J). However, univariate, and multivariate Cox proportional-hazards analyses showed that higher expression of INHBB was not significantly associated with shorter survival time, which requires confirmation in future studies using larger sample sizes. The Western blot and qRT-PCR data of GC tissues from our hospital showed that the INHBB level in GC tissues was higher than that in non-cancer tissues (Fig. 1K-M). The calculation results of Fig. 1M is listed in Supplementary Table S3. Putting side-by-side the above results, it is implied that INHBB may be a worthy predictive factor for GC patients, thus warranting further research in this area. Clinicopathological characteristics information and details of univariate/multivariate analysis of GC cohort are listed in Supplementary Table S4 and S5.

### INHBB promotes GC cell proliferation, migration, and invasion in vitro

**Fig. 2:**
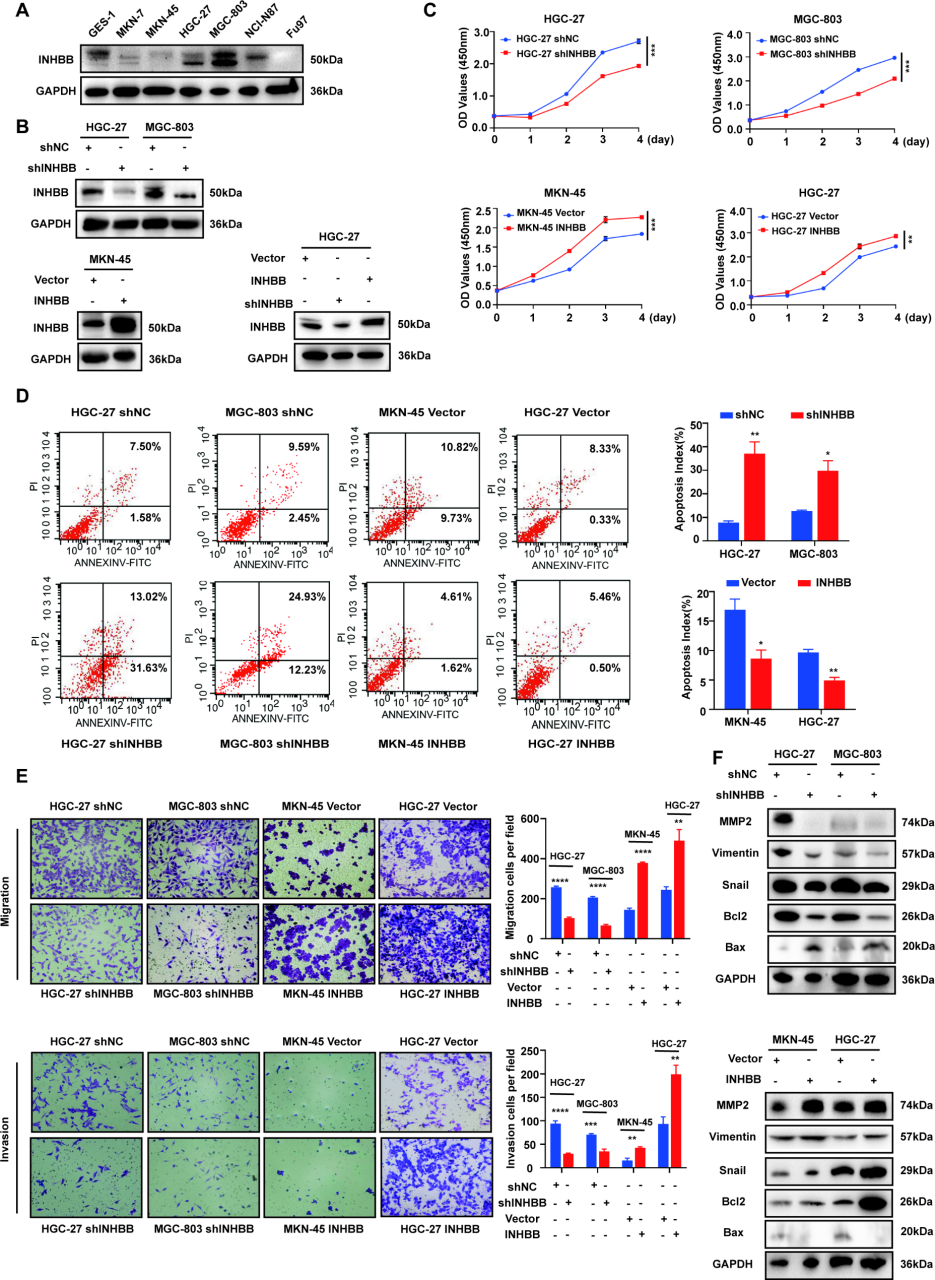
INHBB stimulates GC cell proliferation, migration, and invasion in vitro. **(A)** Protein levels of INHBB was examined by Western blot in GES-1, MKN-7, MKN-45, HGC-27, MGC-803, NCI-N87 and Fu97 cells. **(B)** INHBB expression knockdown by shRNA in HGC-27 (upper) and MGC-803 (upper) and INHBB expression amplification in MKN-45 (lower left) and HGC-27 (lower right) as detected by Western blot. **(C)** Cell proliferation was verified by CCK-8 assay. The value of the absorbance (at 450 nm) was recorded from 0 to 96 h in HGC-27, MGC-803 and MKN-45 infected with shNC, shINHBB, Vector or INHBB amplified lentiviral virus (n = 3). **(D)** Cell apoptosis was detected by flow cytometry in HGC-27, MGC-803 and MKN-45 infected with shNC, shINHBB, Vector or INHBB amplified lentiviral virus (n = 3). **(E)** Cell migration and invasion were detected by transwell assay in HGC-27, MGC-803 and MKN-45 infected with shNC, shINHBB, Vector or INHBB amplified lentiviral virus (n = 3). **(F)** Stable knockdown or overexpression of INHBB affected protein levels of MMP2, Vimentin, Bcl2, Bax and Snail in GC cells. *, P < 0.05; **, P < 0.01; ***, P < 0.001; ****, P < 0.0001

In order to explore the effect of INHBB on the progression of GC, we first detected the expression of INHBB in GC cell lines by qRT-PCR and Western blot, and selected HGC-27, MGC-803 and MKN-45 for loss-or-gain-of-function studies according to the level of endogenous INHBB expression (Fig. 2A, S2A). At the same time, due to the homology between INHBA and INHBB, we excluded the possible influence of INHBA in the cell lines using qRT-PCR analysis. It was found that the expression of INHBA in the three selected cell lines was lower than that in the GES-1 cell line (Fig. S2B). We constructed INHBB knockdown cells lines (HGC-27/shINHBB and MGC-803/shINHBB) by transfection using lentiviral shRNA, and INHBB overexpression cells (MKN-45/INHBB and HGC-27/INHBB) by transfection using INHBB amplified lentivirus (Fig. 2B). Compared with the negative control, CCK-8 assays and colony formation assays showed that INHBB knockdown (HGC-27/shINHBB and MGC-803/shINHBB) significantly inhibited cell proliferation, while INHBB overexpression (MKN-45/INHBB and HGC-27/INHBB) promoted the proliferation of GC cells (Fig. 2C, S2C). The cell apoptosis assay showed that in HGC-27/shINHBB and MGC-803/shINHBB cell lines, the proportion of early and late apoptotic cells increased significantly. In contrast, the overexpression of INHBB reversed this effect and reduced the occurrence of apoptosis (Fig. 2D). In addition, transwell assays also showed that the migration and invasion ability of INHBB-low-expression cells were significantly inhibited compared with INHBB-high-expression cells (Fig. 2E). In HGC-27/shINHBB and MGC-803/shINHBB cells, the levels of pro-apoptosis-related protein Bax increased significantly, while the levels of anti-apoptosis-related protein Bcl2, and epithelial–mesenchymal transition (EMT)-related proteins matrix metalloproteinases 2 (MMP2), Vimentin and Snail decreased significantly. In contrast, MKN-45/INHBB and HGC-27/INHBB cell protein levels showed opposite results (Fig. 2F). Correlation analysis of mRNA levels between INHBB and MMP2, VIM and SNAI1 was carried out, which indicated the presence of significant positive correlations (Fig. S2D-F). These findings suggest that INHBB can promote the progression of GC by reducing cell apoptosis, increasing cell proliferation, migration and invasion ability.

### GC cell-derived activin B induces a CAF phenotype in fibroblasts

**Fig. 3:**
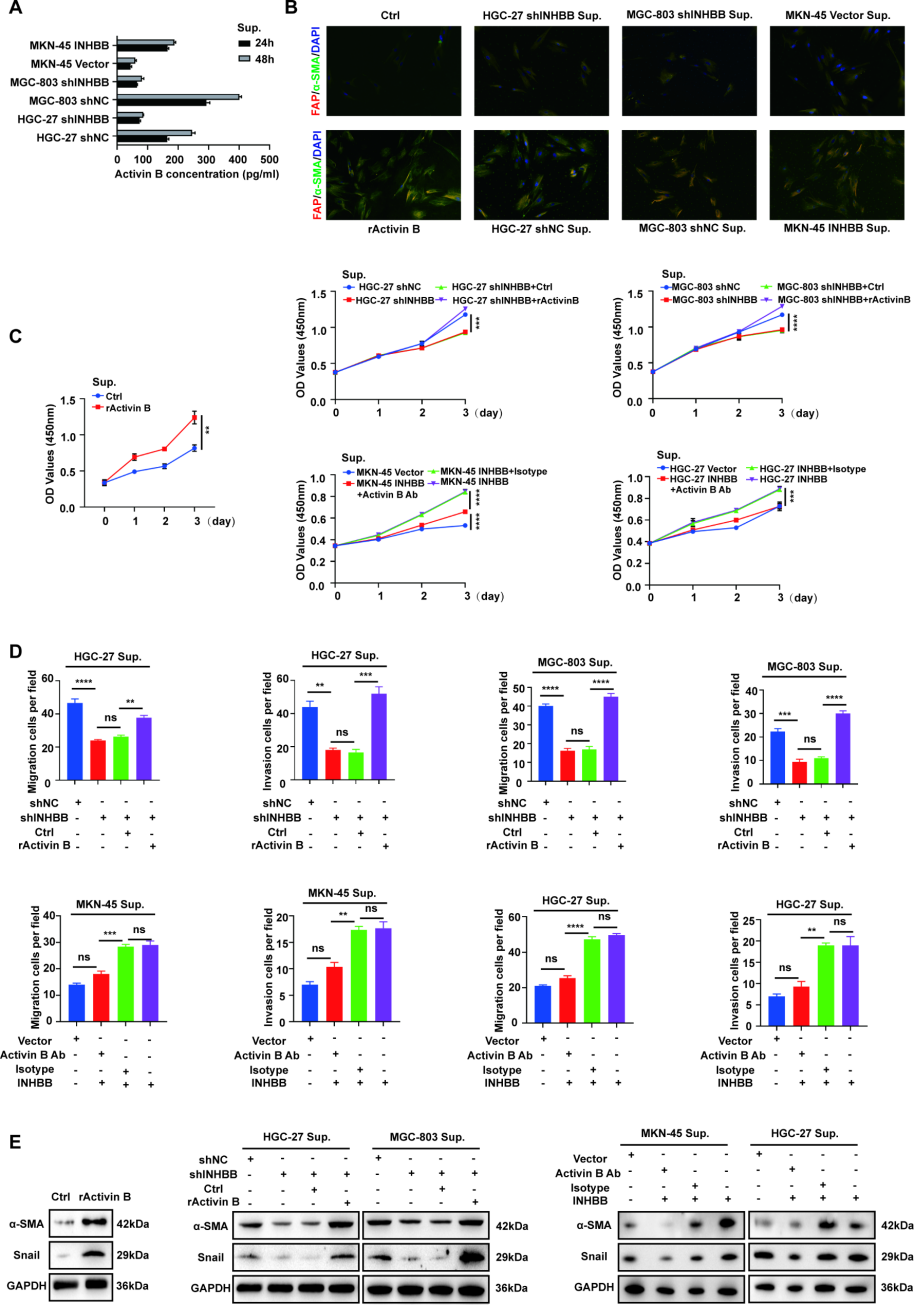
GC cell-derived activin B induces a CAF phenotype in fibroblasts. **(A)** Cell supernatant activin B level was determined by ELISA in different INHBB-expressing GC cells (n = 3). **(B)** Effects of activin B on the normal fibroblast’s protein expression levels of a-SMA and FAP were assessed by immunofluorescence assay. **(C)** Cell proliferation was verified by CCK-8 assay. The value of the absorbance (at 450 nm) was recorded from 0 to 72 h in normal fibroblasts co-cultured with different INHBB-expressing GC cells treated with activin B-neutralizing antibody or exogenous activin B (n = 3). **(D)** Cell migration and invasion were detected by transwell assay in normal fibroblasts co-cultured with different INHBB-expressing GC cells treated with activin B-neutralizing antibody or exogenous activin B (n = 3). **(E)** Western blot detected protein levels of a-SMA and Snail in normal fibroblasts co-cultured with different INHBB-expressing GC cells treated with activin B-neutralizing antibody or exogenous activin B. **, P < 0.01; ***, P < 0.001; ****, P < 0.0001. rActivin B: Recombinant Human Activin B; Sup.: supernatant; Activin B Ab: activin B-neutralizing antibody; Isotype: isotype antibody

Activin B is known as a member of TGF-β family that exists as an INHBB homodimer and plays an important role in renal fibrosis and skin scar hyperplasia. We collected the culture supernatant of cells with different INHBB expression levels for 24 and 48 h, and detected the level of activin B concentration. It was found that the high expression of INHBB in GC cells could lead to the increase of activin B secretion in vitro (Fig. 3A). Since high vitality and motility are hallmarks of CAFs, we examined whether the high expression of INHBB in GC cells could activate fibroblasts by secreting activin B and increase the proliferation and migration and invasion ability of fibroblasts. We first detected the marker genes of CAFs by immunofluorescence staining. The results showed that the expression of alpha-smooth muscle actin (a-SMA) and fibroblast activation protein (FAP) in normal fibroblasts co-cultured with activin B or supernatant of the INHBB-high-expression GC cells (HGC-27 shNC/MGC-803 shNC/MKN-45 INHBB) was significantly higher, while the activation of normal fibroblasts in the INHBB-low-expression group (HGC-27 shINHBB/MGC-803 shINHBB/MKN-45 Vector) was relatively weak (Fig. 3B, S3A). Correlation analysis of mRNA levels between INHBB and a-SMA and FAP based on GEPIA also showed positive correlation results (Fig. S3B-C). The cell proliferation assays showed that the supernatant of INHBB-high-expression GC cells exhibited promotion of the proliferation of normal fibroblasts, which could be weakened by exogenous activin B neutralizing antibody (R&D, MAB659). In contrast, the low expression of INHBB did not significantly improve the proliferation of fibroblasts. However, adding exogenous activin B to this supernatant could significantly promote proliferation (Fig. 3C). In addition, GC cells with different INHBB expression were laid in the lower chamber of the transwell plate, where adding activin B-neutralizing antibody or exogenous activin B allowed us to examine their loss-or-gain-of-function, respectively, in migration and invasion of fibroblasts in the upper chamber. The results showed that the concentration level of activin B in GC cell supernatant was positively correlated with the migration and invasion ability of fibroblasts (Fig. 3D, S3D). Western blot analysis verified that after being stimulated by activin B or co-cultured with INHBB-high-expression GC cells, the CAF biomarker a-SMA and the EMT-related biomarker Snail were significantly increased. Conversely, activin B-neutralizing antibody or INHBB-low-expression GC cell supernatant inhibited the expression of these biomarkers (Fig. 3E).

### GC cell-derived activin B regulates NF-κB activity of fibroblasts through ALK7/TRAF6/TAK1

**Fig. 4:**
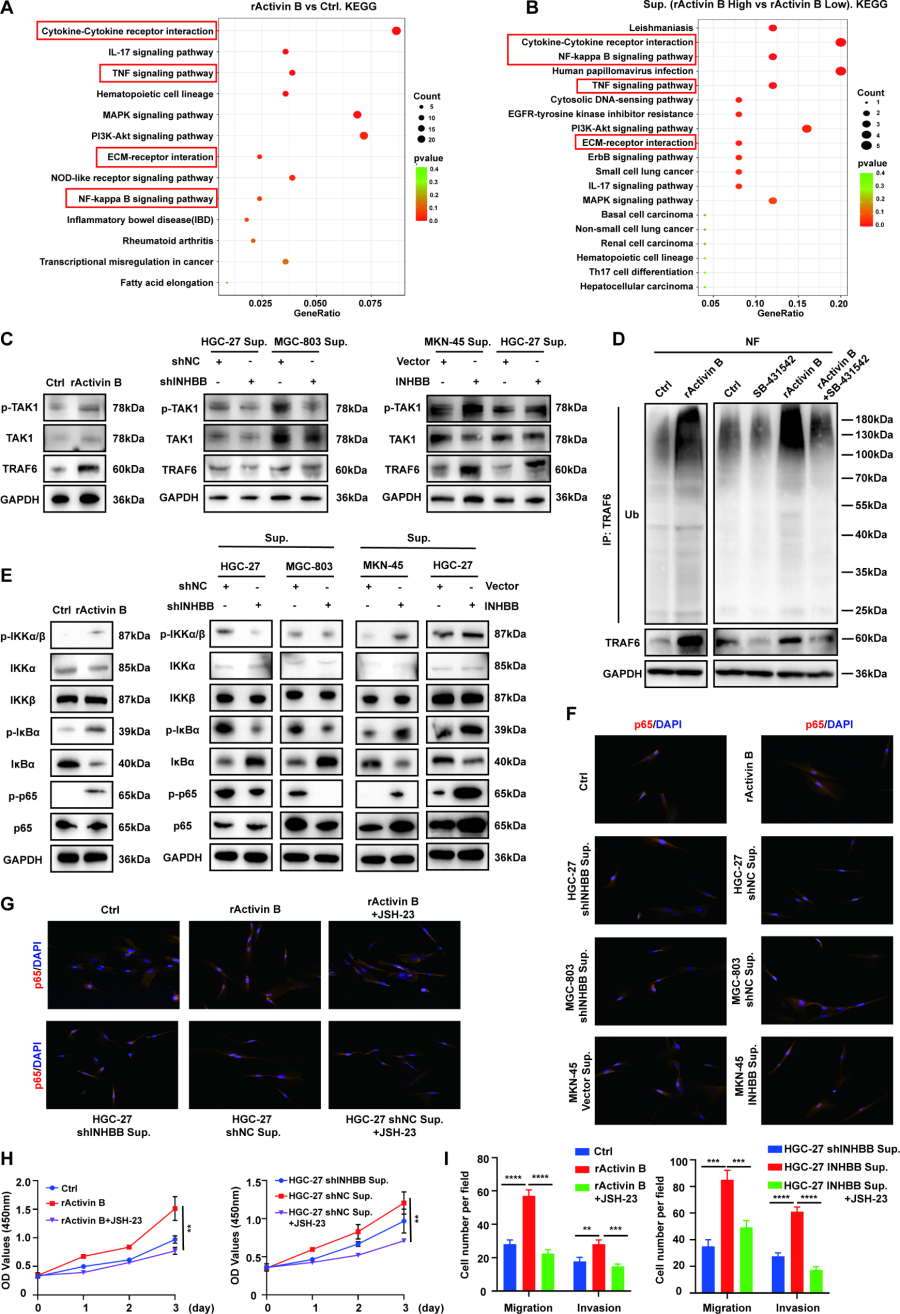
GC cell-derived activin B regulates NF-κB activity of fibroblasts through ALK7/TRAF6/TAK1. **A-B.** KEGG pathway enrichment analysis of significantly differential expression genes (DEGs). **C**. Co-culture with activin B, stable knockdown, or overexpression of INHBB in GC cells affected protein levels of TRAF6 and phosphorylation level TAK1 in normal gastric fibroblasts. **D**. Normal gastric fibroblasts stimulated by activin B or treated by control reagent or SB-431,542. Cell lysates were immunoprecipitated with an anti-TRAF6 antibody and were followed by immunoblotting with anti-ubiquitin antibody. **E**. Western blot analyses of the levels of p-IKKα/β, p-IκBα, p-p65, total IKKα/β, IκBα and p65 in fibroblast co-cultured with activin B or different INHBB-expressing GC cells. **F**. Immunofluorescence assay evaluating the nuclear translocation of p65 in fibroblast co-cultured with activin B or different INHBB expressing GC cells. **G**. Immunofluorescence assay evaluating the effect of JSH-23 to the nuclear translocation of p65 in fibroblasts. **H**. Fibroblasts under high activin B environment were treated with JSH-23 as indicated, and cell proliferation was verified by CCK-8 assay (n = 3). **I**. Fibroblasts under high activin B environment were treated with JSH-23. Transwell assay was used to evaluate cell migration and invasion (n = 3). **, P < 0.01; ***, P < 0.001; ****, P < 0.0001. rActivin B: Recombinant Human Activin B; Sup.: supernatant

To unravel the molecular mechanism by which activin B plays a role in the activation of normal fibroblasts of GC, we performed transcriptome analysis (RNA-Seq) on fibroblasts stimulated by exogenous recombinant human activin B or co-cultured with GC cells (HGC-27 shNC/HGC-27 shINHBB, MGC-803 shNC/MGC-803 shINHBB, MKN-45 Vector/MKN-45 INHBB). Comparing with the control group, the Kyoto Encyclopedia of Genes and Genomes (KEGG) enrichment analysis showed that the Cytokine-Cytokine receptor interaction pathway was significantly enriched. In addition, it was found that high concentration of activin B made genes enriched in TNF signal pathway and NF-κB pathway (Fig. 4A-B). The simultaneous up-regulation of the ECM-receptor interaction pathway suggested a vigorous matrix metabolism. At the same time, according to the gene ontology (GO) enrichment analysis of DEGs, biological processes such as cell migration, cytokine secretion and positive regulation of NF-κB import into nucleus were enriched (Fig. S4A-B). Importantly, based on the public Matrisome database [21], several marker genes, which encode predominantly collagens, ECM-affiliated proteins, and ECM-regulators, were up-regulated in activin B treated fibroblasts (Fig. S4C), providing a possible explanation for the pro-tumorigenic activity of the matrisome and secretome of activin B activated fibroblasts. Correlation analysis of mRNA levels between INHBB and COL10A1 and COL22A1 based on GEPIA also showed positive correlation results (Fig. S4D-E). Tumor necrosis factor receptor-associated factor 6 (TRAF6) is the key activator of TNF signaling pathway and the key upstream regulator of IKK complex, and is one of the important junction proteins of TNF-α and NF-κB signaling pathway. Transforming growth factor β activated kinase 1 (TAK1) is a serine/threonine kinase in the mitogen-activated protein kinase kinase kinase (MAPKKK) family, and is also a recognized direct upstream signal protein for IKK activation [22]. Since the relationship between TRAF6 and the activated kinase TAK1 has been clearly demonstrated in many studies [23–25], we investigated whether TARF6/TAK1 plays a role in the activation of the NF-κB pathway in fibroblasts activated by activin B. Thus, we evaluated whether activin B activated the NF-κB pathway of fibroblasts by regulating TRAF6. Firstly, Western blot results showed that the expression of TRAF6 in GC fibroblasts was increased by activin B stimulation or co-culture with INHBB-high-expression GC cells. Meanwhile, along with increased translation of TRAF6 protein, the phosphorylation level of TAK1 was also up-regulated (Fig. 4C). The immunofluorescence assays provided evidence that TRAF6 and TAK1 were co-located in the cytoplasm of fibroblasts (Fig. S4F). TRAF6 is known to be autoubiquitinated at Lys63, which is essential for activating TAK1 and downstream NF-κB pathway. Thus, we examined whether the increase in TRAF6 expression by activin B could lead to autoubiquitination of TRAF6. ALK7 is the principal type I receptor of activin B and the main receptor for downstream signaling. SB-431,542 (MCE, HY-10,431), a TGF-β receptor kinase inhibitor, is known to have inhibitory effects on ALK4, ALK5 and ALK7 activity, meaning that it can inhibit activin B related receptors [26]. Our results showed that stimulation of activin B promoted TRAF6 autoubiquitination in normal gastric fibroblasts and SB-431,542 inhibited TRAF6 autoubiquitination in fibroblasts (Fig. 4D). Furthermore, we evaluated the expression of Iκ Bα, IKK α/β and p65 in fibroblasts stimulated by activin B or co-cultured with different INHBB-expressing GC cells. The results showed that activin B increased the phosphorylation of p65 and Iκ Bα at Ser32 and Ser36 sites in fibroblasts. Phosphorylated IKK α/β (Ser176/180) has been proved to be indispensable for the activation of NF-κB. Our results also showed that high levels of activin B stimulation increased the level of phosphorylated IKK α/β (Ser176/180) (Fig. 4E). The results of immunofluorescence detection showed that compared with the control groups, the p65 nuclear translocation in fibroblasts under high activin B environment was enhanced (Fig. 4F, S4G). In order to confirm the role of NF-κB pathway in the activation and the enhancement of cell proliferation, and migration and invasion of normal GC fibroblasts promoted by activin B, we used NF-κB/p65 inhibitor, JSH-23 (MCE, HY-13,982), to treat fibroblasts stimulated by Activin B or co-cultured with GC cells (HGC-27 shNC/HGC-27 shINHBB). Our results showed that JSH-23 treatment could inhibit the nuclear translocation of p65 in fibroblasts induced by activin B (Fig. 4G, S4H). Also, it could reverse the cell proliferation, migration and invasion induced by activin B (Fig. 4H-I, S4I).

To further validate the molecular mechanism by which activin B-ALK7 plays a role in the activation of normal fibroblasts of GC, firstly, we detected ALK7 and TRAF6 colocalization in fibroblasts by immunofluorescence assay (Fig. S5A). ALK7 is known to be a transmembrane protein and TRAF6 is known to be a cytoplasmic protein. In fibroblasts stimulated with activin B, compared with the control group, TRAF6 was visualized the local aggregation on the cell membrane, which was consistent with our previous conjecture. We considered transmembrane receptor ALK7 could bind TRAF6 interaction motifs to activate TRAF6 by promoting oligomerization. Additionally, we established normal gastric fibroblasts with ALK7 silence through siRNA (Fig. S5B). In activin B stimulation system with or without transfect with ALK7 siRNA, we detected TRAF6 and TAK1 phosphorylation of fibroblasts by Western blot. The results found that the up-regulation of TRAF6 and phosphorylation of TAK1 activated by activin B was inhibited in ALK7 silent fibroblasts. Furthermore, we evaluated the phosphorylation of IκBα, IKK α/β and p65 in fibroblasts stimulated by activin B with or without transfect with ALK7 siRNA. The results showed that ALK7 silence could reverse the up-regulation of phosphorylation of IκBα, IKK α/β and p65 in fibroblasts induced by activin B. The above results also held in GC cells co-cultured system with or without transfect with ALK7 siRNA (Fig. S5C). Overall, these results indicated that activin B activates and conducts the signaling to fibroblasts through ALK7-TRAF6-TAK1-NF-κB axis.

Considering the high heterogeneity of CAF in GC tumor microenvironment, we typed CAFs induced by activin B. Luo et al. integrated and analyzed CAFs across ten common solid cancer types including gastric cancer through single-cell RNA-seq, identifying their plasticity and interactions with other cell types [27]. According to the overexpressing specific marker genes, the study annotated CAFs into six clusters: cancer-associated myofibroblasts (CAFmyo), inflammatory CAFs (CAFinfla), adipogenic CAFs (CAFadi), endothelial-to-mesenchymal transition CAF (CAFEndMT), peripheral nerve-like CAF (CAFPN) and antigen-presenting CAF (CAFap). According to the previous RNA-seq data, we filtered out 269 up-regulated DEGs with adjust p value < 0.05 and logFC > 1 in fibroblasts treated with activin B. These DEGs were combined with the top differential genes of six CAF clusters to generate Venn diagrams. The results showed that activin B tended to induce fibroblasts into CAFmyo, CAFinfla and CAFEndMT (Fig. S6A-B). The results are preliminary. And we will further isolate primary fibroblasts from patient specimen with different activin B concentration to perform single-cell RNA-seq to confirm this work in the future.

### Activated fibroblasts regulate GC cell function via the IL-1β/p65 pathway

**Fig. 5:**
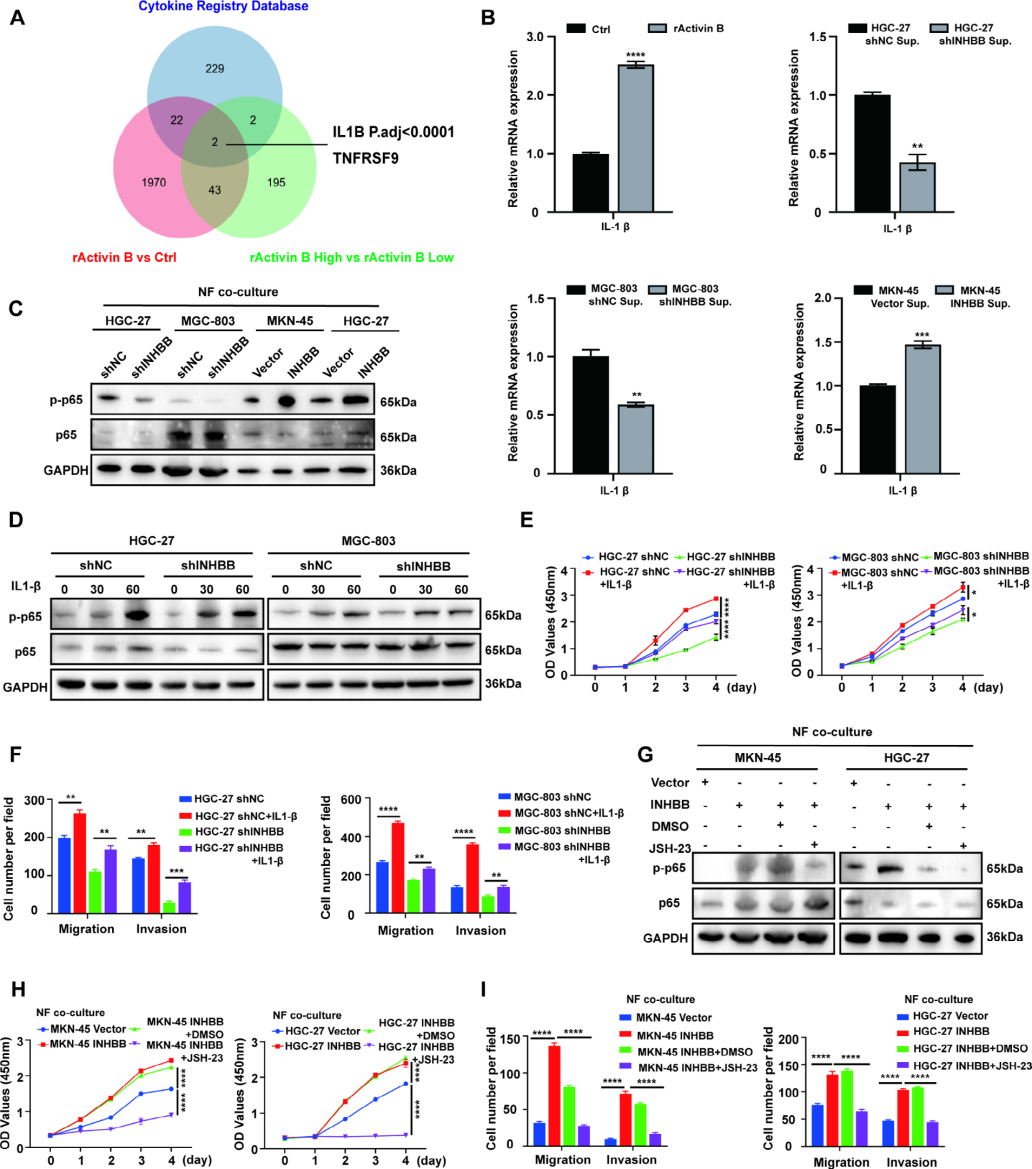
Activated fibroblasts regulate GC cell function via the IL-1β/p65 pathway. **(A)** A Venn diagram summarized the up-regulated cytokine genes in fibroblasts stimulated by exogenous activin B or co-cultured with GC cells (HGC-27 shNC vs. HGC-27 shINHBB; MGC-803 vs. MGC-803 shINHBB; MKN-45 INHBB vs. MKN-45 Vector). **(B)** mRNA levels of IL-1b was examined by qRT-PCR in GC cells (HGC-27 shNC/HGC-27 shINHBB, MGC-803/MGC-803 shINHBB, MKN-45 Vector/MKN-45 INHBB, HGC-27 Vector/HGC-27 INHBB). **(C) **Western blot analyses of the levels of p-p65 and p-65 in GC cells co-cultured with fibroblasts. **(D)** Western blot analyses of the levels of p-p65 and p-65 in GC cells treated with IL-1b for different times. **(E)** Cell proliferation was verified by CCK-8 assay. The value of the absorbance (at 450 nm) was recorded from 0 to 96 h in GC cells treated or untreated with IL-1b (n = 3). **(F)** Cell migration and invasion were detected by transwell assay in GC cells with or without IL-1b co-culture (n = 3). **(G)** Western blot analyses of the levels of p-p65 and p-65 in GC cells treated and untreated with JSH-23 in fibroblasts co-cultured system. **(H)** Cell proliferation was verified by CCK-8 assay. The value of the absorbance (at 450 nm) was recorded from 0 to 96 h in GC cells treated or untreated with JSH-23 in fibroblasts co-cultured system (n = 3). **(I) **Cell migration and invasion were detected by transwell assay in GC cells in fibroblasts co-cultured system with or without JSH-23 co-culture (n = 3). *, P < 0.05; **, P < 0.01; ***, P < 0.001; ****, P < 0.0001. rActivin B: Recombinant Human Activin B; Sup.: supernatant

Previous studies have shown that NF-κB activity induces the secretion of many inflammatory factors, which are closely related to the progression of GC [28, 29]. Using combination of Cytokine Registry Database (https://www.immport.org/resources/cytokineRegistry) and up-regulated DEGs from RNA-seq, we found that IL-1β was significantly enriched (Fig. 5A). Therefore, we evaluated the expression of NF-κB dependent cytokine IL-1β through qRT-PCR. Our results showed that high concentration of activin B indeed stimulated the expression of NF-κB target gene IL-1β in fibroblasts (Fig. 5B). IL-1β has been widely studied in GC and chronic gastric diseases, and it is confirmed that it is closely related to the development of GC and chronic inflammation [30, 31]. Overall survival analysis of IL-1β between high- and low-expression GC patients based on GEPIA also showed positive results (Fig. S7A). Additionally, this pro-inflammatory factor has been generally recognized as the upstream activator of p65, a key component of the canonical NF-κB pathway [32, 33]. We found that overexpression of INHBB increased the phosphorylation of p65 in GC cells in the co-cultured environment with normal gastric fibroblasts. In contrast, INHBB knockdown reduced the phosphorylation level of p65 in GC cells in co-cultured environment in normal fibroblasts (Fig. 5C). However, stimulation of GC cells with activin B alone did not cause p65 phosphorylation (Fig. S7B). In order to confirm the role of p-p65 in promoting the proliferation, migration, and invasion of GC cells, we firstly used exogenous IL-1β (MCE, HY-P78459) to act on HGC-27 and MGC-803 control cells and INHBB knockdown cells, where we found that IL-1β could promote the phosphorylation of p65 in GC cells (Fig. 5D), thus inducing the simultaneous increase of cell proliferation, migration, and invasion (Fig. 5E-F, S7C). In addition, we used a p65 phosphorylation inhibitor (JSH-23) to act on INHBB-overexpressing MKN-45 and HGC-27 GC cells co-cultured with fibroblasts. It was found that JSH-23 inhibited the phosphorylation of p65 (Fig. 5G) and reversed the cell proliferation, migration and invasion induced by the overexpression of INHBB in the co-cultured environment with fibroblasts (Fig. 5H-I, S7D). Considering a potential effect on GC cells may exist of JSH-23, we established normal gastric fibroblasts with IL-1β silence through siRNA (Fig. S8A). In fibroblasts co-cultured system with or without transfect with IL-1β siRNA, we detected p65 phosphorylation of MKN-45 and HGC-27 by Western blot. The results found that IL-1β silence inhibited the phosphorylation of p65 in INHBB overexpressing GC cells (Fig. S8B). CCK-8 and transwell assays further demonstrated that IL-1β silence of fibroblasts could reverse the GC cell proliferation, migration and invasion induced by the high concentration of IL-1β in the co-cultured environment with fibroblasts (Fig. S8C-E). Overall, these results indicated that activin B from GC cells promoted NF-κB activation and IL-1β expression in fibroblasts. The secretion of IL-1β in fibroblasts could in turn promote the proliferation, migration and invasion of GC cells, so as to promote cancer progression.

### p65 directly binds to the INHBB promoter and induces INHBB expression in GC cells

**Fig. 6:**
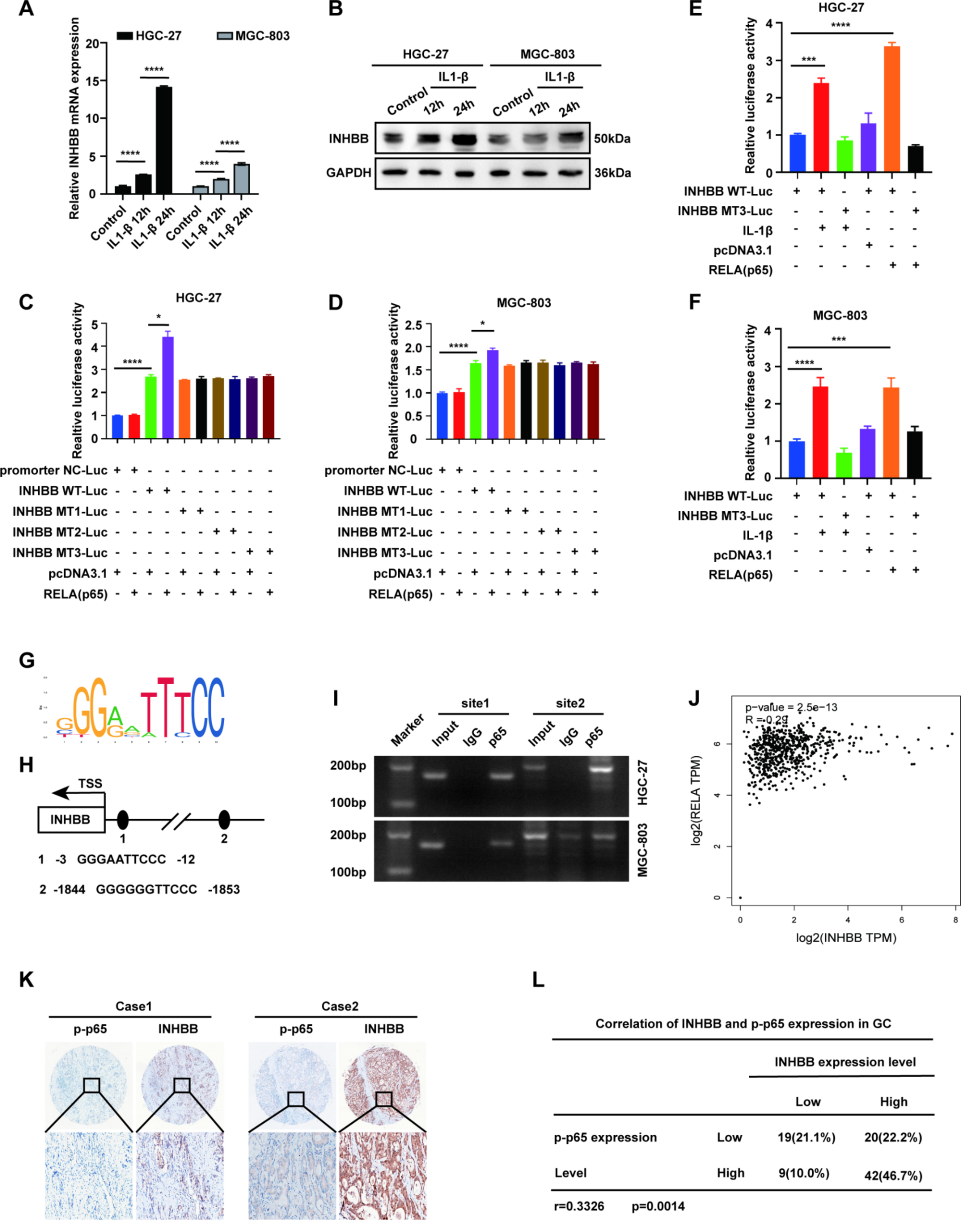
p65 directly binds to the INHBB promoter and induces INHBB expression in GC cells. **(A) **RT-PCR analysis was conducted to determine the expression levels of INHBB mRNA in cells treated with IL-1b. **(B) **Western blot analysis was conducted to determine the expression levels of INHBB protein in cells treated with IL-1b. **C-D.** Luciferase reporter vectors containing wild-type or mutants INHBB and p65 or the vector were transfected into HGC-27 and MGC-803. Luciferase activities were measured using luciferase assays. **E-F.** The wild type and mutants INHBB luciferase reporter vectors were transfected into HGC-27 and MGC-803 cells. Treatment with IL-1b or transfection with p65 or pcDNA3.1, the corresponding relative luciferase activity was determined by luciferase assays (n = 3). **G**. p65 binding motif. **H**. Jasper transcription factor binding site prediction supplied two potential p65-binding sites of INHBB. **I.** Agarose electrophoresis performed to verify p65 binding to the INHBB promoter. **J.** Correlation analysis of mRNA levels between INHBB and p65 based on TCGA database using GEPIA. **K**. Representative IHC staining images of INHBB and p65 in GC tissues (n = 90). **L**. The correlation between INHBB and p-p65 levels in 90 GC tissues was analyzed. ***, P < 0.001; ****, P < 0.0001. p65: RELA.

Our above results suggested that IL-1β from GC microenvironment could promote the proliferation, migration and invasion of GC cells by activating p65. We tried to identify whether p65 can directly regulate the expression of INHBB in GC cells through nuclear transcription. For this purpose, we first evaluated the expression of INHBB in GC cells stimulated by exogenous IL-1β. QRT-PCR and Western blot results showed that the expression of INHBB induced by IL-1β increased in a time-dependent manner (Fig. 6A-B). In the next step, we tried to determine whether p65 can directly regulate the expression of INHBB in GC cells. In order to evaluate the upstream regulation mechanism of INHBB in GC, we carried out double luciferase report assay, and constructed reporter gene plasmids containing wild-type INHBB promoter (INHBB WT) or mutant INHBB promoter (INHBB MT) containing mutant p65 binding site to determine the regulatory elements controlling INHBB transcription. We found that overexpression of p65 increased the activity of INHBB WT promoter in HGC-27 and MGC-803, but did not significantly increase the activity of INHBB MT promoter (Fig. 6C-D). In addition, we found that IL-1β increased the activity of INHBB WT promoter in HGC-27 and MGC-803 cells (Fig. 6E-F). The binding sites of p65 and INHBB according to the public database transcription factor prediction (JASPAR Version 3.0) are shown in Fig. 6G-H. In order to verify whether INHBB is a direct target of p65 in intact cells, we performed CHIP assay. CHIP results showed that p65 could directly bind to INHBB promoter (Fig. 6I). Correlation analysis of mRNA levels between INHBB and p65 based on GEPIA also showed positive correlation results (Fig. 6J).

In order to further explore the clinical application of p65 and INHBB, we evaluated the relationship between INHBB and p-p65 in human GC tissue. Our results showed that there was a significant positive correlation between the expression of INHBB and the phosphorylation of p65 in GC tissues (Person r = 0.3326, *P* = 0.0014) (Fig. 6K-L). To sum up, in GC, there is a positive feedback regulation circuit between the p65/IL-1β in fibroblasts and p65/INHBB/activin B in GC cells.

### Elevated circulating activin B associates with gastric cancer

**Fig. 7:**
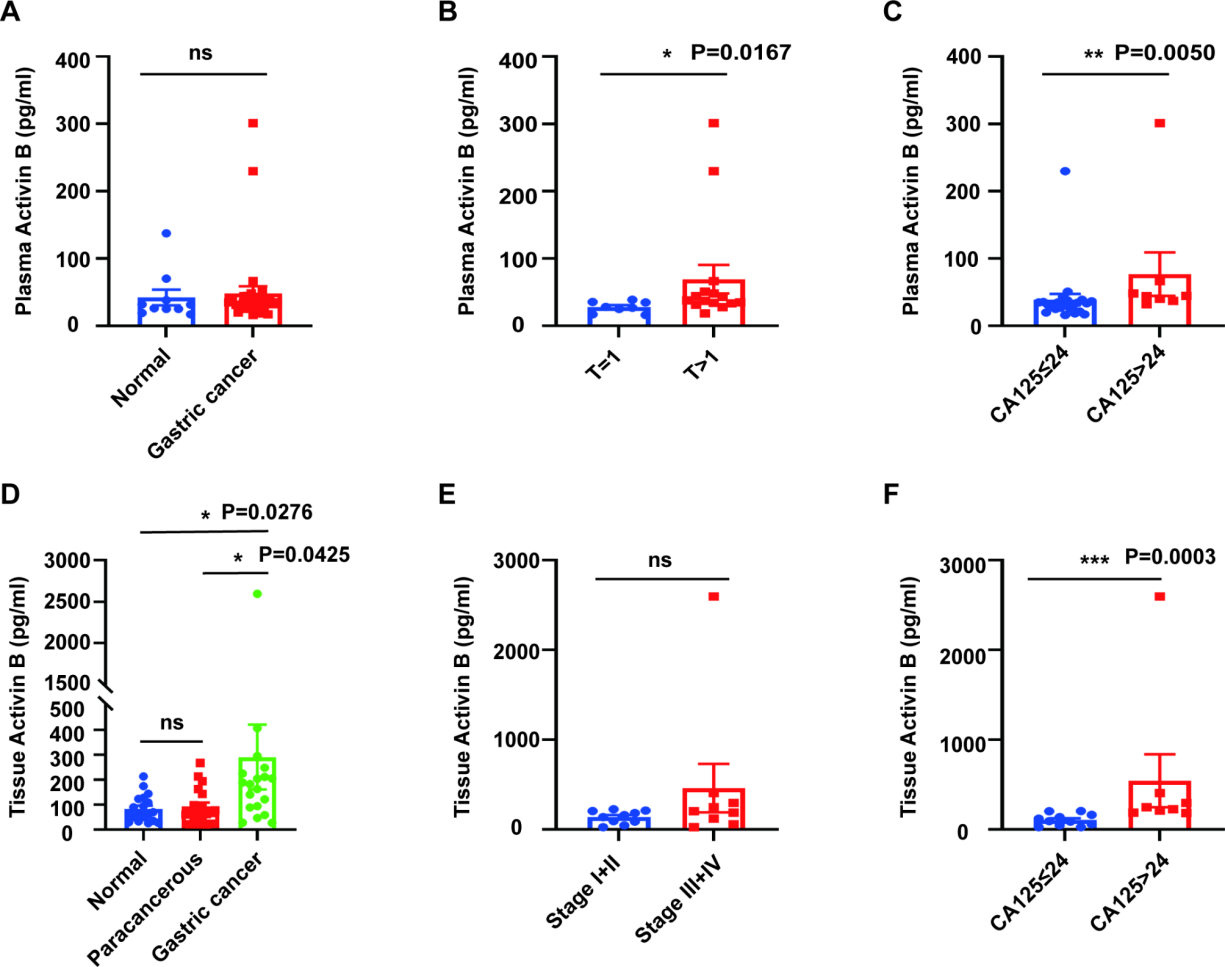
Elevated circulating activin B associates with GC. **A. **Boxplot representing the plasma activin B level between GC patients and healthy volunteers (n = 42). **B-C.** Boxplots representing the plasma activin B level in the different clinicopathological features of GC patients (n = 32). **D**. Boxplot representing the tissue activin B level among gastric cancer, matched normal tissues and matched paracancerous lesions (n = 19). **E-F**. Boxplots representing the tissue activin B level in the different clinicopathological features of GC patients (n = 19). *, P < 0.05; **, P < 0.01; ***, P < 0.001

Plasma activin B levels were measured in 32 patients with GC and 10 healthy individuals. There was no between-group difference in activin B when comparing normal controls and GC patients (median level of activin B in GC patients = 34.90 pg/mL; median level of activin B in healthy individuals = 28.85 pg/mL; P = 0.3543) (Fig. 7A). In addition, according to the clinicopathological characteristics of patients, we found that activin B showed no statistically significant difference with respect to age, sex, N, M, AJCC stages and most tumor biomarkers (CEA, CA199, CA724 and AFP) of GC patients (Fig. S9A-I). Nevertheless, when comparing GC patients with T stage equal or earlier than T1 stage with those with T stage later than T1, it was found that the plasma concentration of activin B was higher in the latter group (P = 0.0167) (Fig. 7B). Furthermore, we also found that there was a correlation between plasma CA125 concentration and activin B, and the plasma activin B concentration of patients with abnormally high CA125 was significantly higher (P = 0.0050) (Fig. 7C). To further validate this work, we collected the primary lesion samples and their matched adjacent samples and normal samples from 19 patients who underwent radical resection of GC in our hospital for tissue ELISA. The results additionally demonstrated that activin B was highly expressed in GC tissues compared to the matched adjacent samples and normal samples, and its concentration was positively correlated with plasma CA125 in GC patients (Fig. 7D, F). There were mainly T3 stage tumors, therefore no comparison of activin B levels in tissues of different T stages. Tissue activin B showed no statistically significant difference with respect to age, sex, AJCC stages and most tumor biomarkers (CEA, CA199, CA724 and AFP) of GC patients (Fig. 7E; S9J-O). Thus, our results proved that there was a certain relationship between an increasing trend in activin B and the higher concentration of plasma CA125 in GC patients, which requires confirmation in future studies using larger sample sizes. The cut-off values of all tumor biomarkers were determined according to the standards of the Laboratory Department of Ruijin Hospital.

### INHBB promotes gastric cancer progression in vivo

**Fig. 8:**
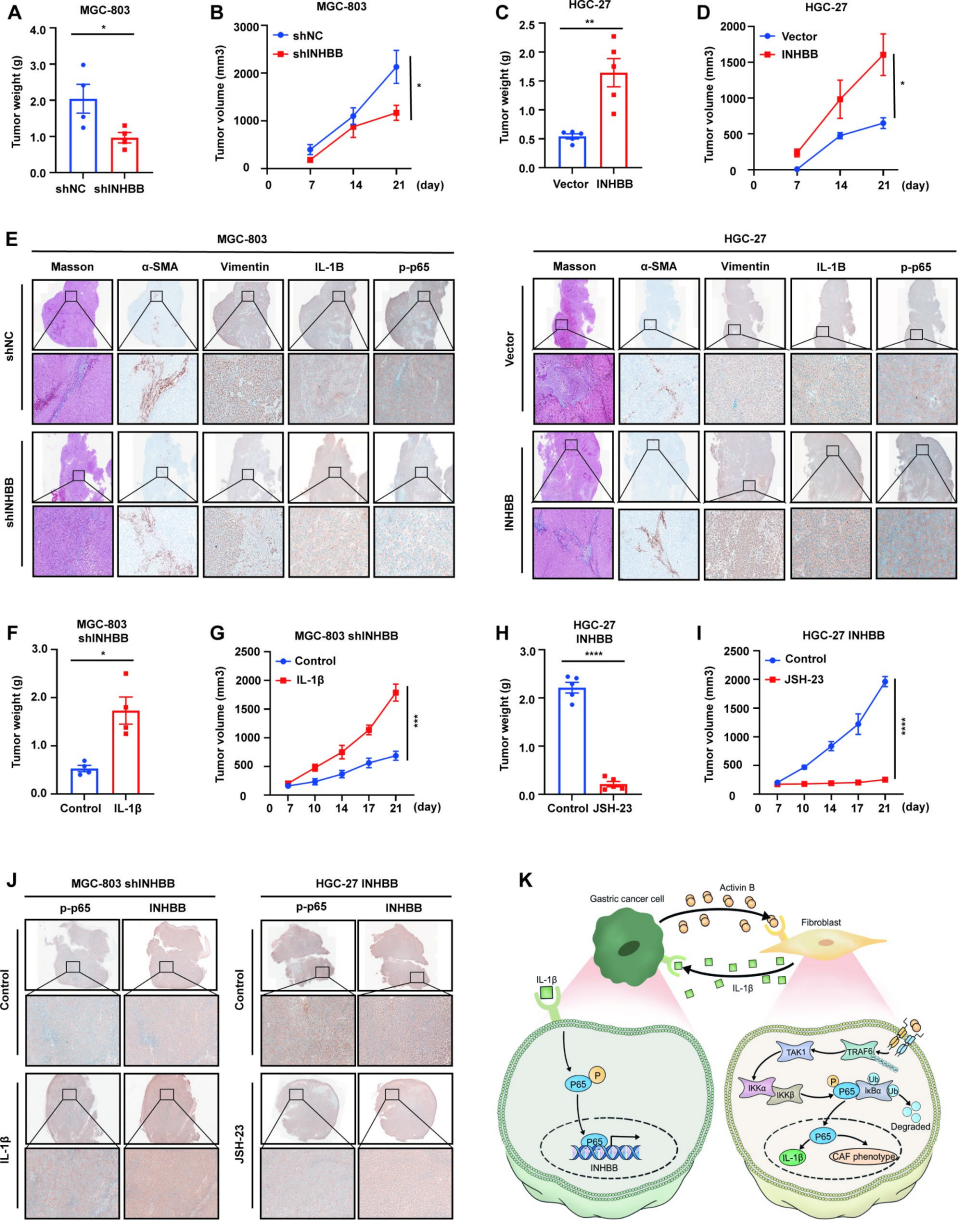
INHBB promotes GC progression in vivo. **(A)** The quantitative analysis of tumor weight from MGC-803 cells with stable INHBB knockdown or vehicle control (n = 4). **(B)** The growth curve of tumor volume from MGC-803 cells with stable INHBB knockdown or vehicle control (n = 4). **(C)** The quantitative analysis of tumor weight from HGC-27 cells with stable INHBB overexpressing or vehicle control (n = 5). **(D)** The growth curve of tumor volume from HGC-27 cells with stable INHBB overexpressing or vehicle control (n = 5). **(E)** The expression of collagen, a-SMA, Vimentin, IL-1B and p-p65 were evaluated by IHC in tissues of xenograft. **(F) **The quantitative analysis of tumor weight from MGC-803 shINHBB cells with IL-1b treatment or control treatment (n = 4). **(G)** The growth curve of tumor volume from MGC-803 shINHBB cells with IL-1b treatment or control treatment (n = 4). **(H)** The quantitative analysis of tumor weight from HGC-27 INHBB cells with JSH-23 treatment or control treatment (n = 5). **(I) **The growth curve of tumor volume from HGC-27 INHBB cells with JSH-23 treatment or control treatment (n = 5). **(J)** The expression of p-p65 and INHBB in tissues of xenograft. **K.** Diagrammatic presentation of the INHBB/NF-kB/IL-1b positive regulatory feedback loop between GC cells and fibroblasts that can promote GC progression. Activin B, the dimer structure formed by INHBB subunit, activates NF-kB pathway by controlling the autoubiquitination of TRAF6 and inducing TAK1 phosphorylation in fibroblasts. Activation of NF-kB releases IL-1b, which facilitates the phosphorylation of p65 and increases GC cells proliferation and invasion. Furthermore, p65 directly promotes INHBB transcription and induces INHBB expression, establishing a positive- feedback loop in GC microenvironment. *, P < 0.05; **, P < 0.01; ***, P < 0.001; ****, P < 0.0001

To further clarify the effects of INHBB in GC tumor microenvironment in vitro, we next verified the roles of INHBB on GC tumorigenicity in vivo. INHBB-overexpressing GC cells or INHBB-knockdown GC cells were subcutaneously inoculated with normal gastric fibroblasts to the right armpit of nude mice. In keeping with our above data, mice inoculated with INHBB-knockdown GC cells showed significant inhibition of tumor growth and decrease of tumor burden compared with those of tumors derived from control cells (Fig. 8A-B, S10A). Conversely, mice bearing INHBB-overexpressing cells had significantly larger tumors compared to mice bearing control cells (Fig. 8C-D, S10B). We verified the expression of INHBB in mice xenografts. (Fig. S10C). Furthermore, our results showed that in INHBB-derived overexpressing tumors, the areas of collagen-positive or α-SMA-positive fibroblasts increased significantly, indicating a profibrotic fibroblast phenotype. Together with prophase data, the number of Vimentin, IL-1β, and p-p65 positive GC cells was also higher in INHBB-overexpressing mice xenografts compared to that of the control groups (Fig. 8E). IHC statistical analyses were presented in Fig. S10D. We next evaluated the efficacy of IL-1b and JSH-23 in vivo. IL-1b (100 ng per mouse; in tumor; twice a week; for 2 weeks) significantly reversed the growth of subcutaneous INHBB-knockdown MGC-803 xenografts (Fig. 8F-G, S10E). Also, JSH-23 (1 mg/kg; orally administered; daily; for 2 weeks) significantly suppressed the growth of subcutaneous INHBB-overexpressing HGC-27 xenografts (Fig. 8H-I, S10F). IHC results showed that IL-1b promoted p65 phosphorylation and up-regulated INHBB expression in mice xenografts from INHBB-knockdown MGC-803 cells when compared to shNC cells. On the contrary, JSH-23 inhibition of p65 phosphorylation weakened the expression of INHBB in mice xenografts from INHBB-overexpressing HGC-27 cells (Fig. 8J). IHC statistical analyses were presented in Fig. S10G. The administration design is presented in Fig. S10H.

## Discussion

Our study confirmed that INHBB was up-regulated in GC, which could promote GC cell proliferation, migration, and invasion. In addition, GC cells overexpressing INHBB increased the activity of NF-κB pathway in normal gastric fibroblasts by secreting activin B, and induced the reprogramming of fibroblasts. The activation of NF-κB leads to the release of inflammatory cytokine IL-1β, which leads to the structural activation of p65 in GC. Interestingly, we also found that p65 could cause an increase in INHBB transcriptional expression, which indicated that there is a positive feedback loop in GC. Our study showed that activin B is a major player in activating fibroblasts into pro-tumorigenic CAFs, and for the first time proposed an underlying molecular mechanism for INHBB in GC in combination with tumor microenvironment.

Changes in the function of cell components in the tumor microenvironment led to phenotypic variation and ultimately affect the tumor outcome. Fibrosis and inflammation are both microenvironment phenotypes that are closely related to tumor progression, thus promoting the continuous growth of the tumor [3]. The activation of resident normal fibroblasts mediates the transformation of cells to CAFs. An increase in proliferation and infiltration also involves the excessive deposition of ECM, thus providing a physical scaffold for the migration of tumor cells [34]. In the present study, we found that activin B can activate the NF-κB pathway of fibroblasts, promote the activation of fibroblasts, induce their proliferation, and up-regulate the migration and invasion phenotype. Simultaneously, it also induced the expression of CAF markers, including cytokines and ECM components. The activation of NF-κB plays an important role in the process of disease fibrosis. In chronic lung diseases, NF-κB can induce the process of pulmonary fibrosis by mediating the up-regulation of NLRP3 or NOX4 [35, 36]. In a model of renal interstitial fibrosis, NF-κB can induce EMT through the secretion of inflammatory cytokines [37]. In the present study, we found that the pro-inflammatory cytokine IL-1β showed a significantly increased production in normal gastric fibroblasts activated by activin B. Previous studies have shown that the inflammatory microenvironment is closely related to the occurrence and development of GC and the mutation of carcinogenic genes, where some inflammatory factors have been proved to be independent risk factors for GC [38]. Among these, IL-1β has been shown to increase the proliferation of GC cells through receptor-mediated tyrosine kinase pathway [39]. In the clinical setting, IL-1β polymorphisms were found to be closely associated with an increased risk of GC [40]. Therefore, the increase of IL-1β secretion of fibroblasts induced by NF-κB activation may promote the progression of GC.

As an important component of NF-κB transcription factor, p65 plays an important role in inflammation, immunity, cell proliferation and apoptosis. In addition, previous studies have shown that p65 is highly expressed in tumor tissues of patients with GC, which is positively correlated with higher degrees of tumor invasion, lymph node metastasis and distant metastasis [41]. In addition, overexpression of p-p65 in tumor tissue has been found to be associated with poor prognosis of GC patients [42]. As a part of the inflammatory tumor microenvironment, IL-1β can participate in the activation of NF-κB canonical pathway either directly (via p65) or in combination with other inflammatory factors, induce the positive feedback expression of various chemokines and inflammatory factors, and build a complex dynamic pro-tumor cycle [43]. In the present study, we found that IL-1β derived from gastric fibroblasts activated by activin B could activate the phosphorylation of p65 in GC cells under co-culture conditions. As a part of NF-κB transcription factor, p65 can directly bind to the INHBB promoter and promote the transcription of INHBB. Accordingly, we believe that the positive feedback loop between GC cell p65/INHBB/activin B and fibroblast p65/IL-1β controls the progression of GC. Furthermore, previous studies have shown that INHBA, which is homologous to INHBB in tumor cells, can partially mediate the activation of CAF in breast and ovarian tumor models. This activation is accomplished by INHBA-induced DNA damage or adrenergic signaling in tumor cells. [44–46]. Thus, our results brought corroborative evidence in the important role of INHBB as a CAF inducer.

In recent years, the microenvironment-targeted treatment of GC has begun to rise, including using various new drugs targeting FGFR [47], VEGFR [48, 49], MMP9 [50] and TGF-β1 [51] for reshaping the matrix microenvironment [52]. Despite being a preliminary examination on showing the possible mechanism of INHBB’s impact on the microenvironment of GC, our study indicates that targeting fibroblast-cancer cell crosstalk is a promising alternative in GC clinical treatment by providing a well-explained possibility for the mechanism in place. GC progression is a huge and extremely complex interactive network, and there is still a long way to go to translate experimental research to clinical applications.

In conclusion, our research revealed that INHBB creates a local tumor-promoting inflammatory environment between GC cells and fibroblasts, thus providing new evidence for the role of INHBB in the occurrence and development of GC (Fig. 8K). We believe these findings have practical significance, which may deepen our understanding regarding the effects of INHBB in GC progression and treatment.

### Electronic supplementary material

Below is the link to the electronic supplementary material.

Supplementary Material 1

Supplementary Material 2

Supplementary Material 3

## Data Availability

The datasets used/or analyzed during the study are available from the corresponding author on reasonable request.

## Abbreviations

GC: gastric cancer
CAFs: cancer-associated fibroblasts
INHBB: inhibin β subunit
ECM: extracellular matrix
IHC: immunohistochemistry
CHIP: chromatin immunoprecipitation
IP: immunoprecipitation
OS: overall survival
TCGA: The Cancer Genome Atlas Database
DFS: disease-free survival
EMT: epithelial–mesenchymal transition
MMP2: matrix metalloproteinases 2
α-SMA: alpha-smooth muscle actin
FAP: fibroblast activation protein
KEGG: the Kyoto Encyclopedia of Genes and Genomes
GO: the gene ontology
DEGs: differential expression genes
TRAF6: tumor necrosis factor receptor-associated factor 6
TAK1: transforming growth factor β activated kinase 1
MAPKKK: mitogen-activated protein kinase kinase kinase
