# Supplementary material for: Paracrine activin B-NF-κB signaling shapes an inflammatory tumor microenvironment in gastric cancer via fibroblast reprogramming
Source: J Exp Clin Cancer Res. 2023 Oct 19;42:269. doi: 10.1186/s13046-023-02861-4 (PMC10585924; doi:10.1186/s13046-023-02861-4)
Supplement: Supplementary file 3 — Supplementary Material 3 [file 13046_2023_2861_MOESM3_ESM.docx]

**Supplementary tables**

**Supplementary Table 1.** Antibodies used in this study

| Antibody | Catalog | Dilution | Company |
| --- | --- | --- | --- |
| **For Western blotting** | | | |
| INHBB | PK76232 | 1:1000 | Abmart |
| MMP2 | T57164 | 1:1000 | Abmart |
| BCL2 | 15071 | 1:1000 | CST |
| BAX | 41162 | 1:1000 | CST |
| Vimentin | 5741 | 1:1000 | CST |
| α-SMA | ab5694 | 1:1000 | Abcam |
| Snail | A5243 | 1:1000 | Abclone |
| TRAF6 | T55175 | 1:1000 | Abmart |
| TAK1 | 12330-2-AP | 1:1000 | Proteintech |
| P-TAK1 | 4537 | 1:1000 | CST |
| IL-1B | MK56352 | 1:1000 | Abmart |
| NF-κB Pathway Antibody Sampler Kit | 9936 | 1:1000 | CST |
| GAPDH | 5174S | 1:1000 | CST |
| Secondary antibody | 7076S | 1:5000 | CST |
| Secondary antibody | 7074S | 1:5000 | CST |
| **For Immunohistochemistry** | | | |
| INHBB | PK76232 | 1:100 | Abmart |
| P-P65 | TP56372 | 1:50 | Abmart |
| Vimentin | 5741 | 1:100 | CST |
| α-SMA | ab5694 | 1:100 | Abcam |
| Secondary antibody | Envision kit (HRP, rabbit/mouse, DAB+) | Ready-to-  use | DAKO |
| **For Immunofluorescence staining** | | | |
| FAP | 66562 | 1:100 | CST |
| α-SMA | M0874 | 1:200 | DAKO |
| P65 | 8242 | 1:250 | CST |
| TAK1 | 12330-2-AP | 1:200 | Proteintech |
| TRAF6 | T55175 | 1:200 | Abmart |
| ALK7 | BS-5777R | 1:100 | Bioss |
| DAPI | C1002 | 1:1000 | Beyotime |
| Secondary antibody | Alexa Fluor 555  anti-rabbit IgG | 1:50 | Invitrogen |
| Secondary antibody | Alexa Fluor 488  anti-mouse IgG | 1:50 | Invitrogen |
| Secondary antibody | Alexa Fluor 488  anti-rabbit IgG | 1:50 | Invitrogen |

**Supplementary Table 2.** The sequences of gene-specific primers used for qRT-PCR, vector constructs and ChIP assay

| Gene name | Forward (5’-3’) | Reverse (5’-3’) |
| --- | --- | --- |
| **Primers for qRT-PCR** | | |
| INHBB | GTGAAGCGGCACATCTTGAG | GCGAAGCTGATGATTTCGGAAAC |
| INHBA | CCTCCCAAAGGATGTACCCAA | CTCTATCTCCACATACCCGTTCT |
| IL-1B | GGCCCTAAACAGATGAAGTGC | CCAGCATCTTCCTCAGCTTG |
| GAPDH | AGAAGGCTGGGGCTCATTTG | AGGGGCCATCCACAGTCTTC |
| **Primers for vector constructs** | | |
| H_INHBB promoter (-2000 to +50) WT | GTACCGAGCTCTTACGCGTG | AGCTTACTTAGATCGCAGATCTCGA |
| H_INHBBpromoter (-2000 to +50) MT1 | GTACCGAGCTCTTACGCGTG | AGCTTACTTAGATCGCAGATCTCGA |
| H_INHBBpromoter (-2000 to +50) MT2 | GTACCGAGCTCTTACGCGTG | AGCTTACTTAGATCGCAGATCTCGA |
| H_INHBBpromoter (-2000 to +50) MT3 | GTACCGAGCTCTTACGCGTG | AGCTTACTTAGATCGCAGATCTCGA |
| H_RELA | CTTGGTACCGAGCTCGGATCCGCCACCATGGACGAACTGTTC | AACGGGCCCTCTAGACTCGAGTTAGGAGCTGATCTGACTCAGCAGG |
| **Primers for ChIP** | | |
| INHBB (promoter for RELA)-1 | AAAGTGGGAATTCCCTGGGGGT | TGGAGGGCGGGAAGGCAGTA |
| INHBB (promoter for RELA)-2 | GGAAAGGATGGGGAGAAGGCTGC | AGGGCTGCCGCCGGCTCG |

**Supplementary Table 3.** The calculation results of tissue PCR

| Sample number | INHBB mRNA expression Log (Tumor/Normal,10) | Sample number | INHBB mRNA expression  Log (Tumor/Normal,10) |
| --- | --- | --- | --- |
| Sample 1. | -4.81 | Sample 11. | 0.59 |
| Sample 2. | -0.84 | Sample 12. | 0.69 |
| Sample 3. | -0.45 | Sample 13. | 0.74 |
| Sample 4. | -0.17 | Sample 14. | 0.91 |
| Sample 5. | -0.15 | Sample 15. | 1.10 |
| Sample 6. | 0.12 | Sample 16. | 1.35 |
| Sample 7. | 0.16 | Sample 17. | 1.42 |
| Sample 8. | 0.23 | Sample 18. | 1.43 |
| Sample 9. | 0.34 | Sample 19. | 1.50 |
| Sample 10. | 0.55 | Sample 20. | 1.56 |

**Supplementary Table 4.** Correlation between INHBB levels in GC patients and their clinicopathological characteristics

| Clinicopathological features | Low expression  N (%) | High expression  N (%) | p value |
| --- | --- | --- | --- |
| N | 28 (31.1%) | 62 (68.9%) |  |
| Gender |  |  | 0.484 |
| Male | 18 (20%) | 35 (38.9%) |  |
| Female | 10 (11.1%) | 27 (30%) |  |
| Age |  |  | 0.324 |
| ≤ 65 | 15 (16.7%) | 40 (44.4%) |  |
| >65 | 13 (14.4%) | 22 (24.4%) |  |
| T.stage |  |  | **< 0.001***** |
| 1 | 3 (3.3%) | 0 (0%) |  |
| 2 | 8 (8.9%) | 3 (3.3%) |  |
| 3 | 13 (14.4%) | 40 (44.4%) |  |
| 4 | 4 (4.4%) | 19 (21.1%) |  |
| N.stage |  |  | 0.519 |
| 0 | 9 (10%) | 14 (15.6%) |  |
| 1 | 5 (5.6%) | 7 (7.8%) |  |
| 2 | 4 (4.4%) | 14 (15.6%) |  |
| 3 | 10 (11.1%) | 27 (30%) |  |
| M.stage |  |  | 1.000 |
| 0 | 27 (30%) | 59 (65.6%) |  |
| 1 | 1 (1.1%) | 3 (3.3%) |  |
| Clinical stage |  |  | **0.011*** |
| I | 7 (7.8%) | 2 (2.2%) |  |
| II | 9 (10%) | 18 (20%) |  |
| III | 11 (12.2%) | 39 (43.3%) |  |
| IV | 1 (1.1%) | 3 (3.3%) |  |
| Pathologic differentiation |  |  | 0.187 |
| Well/Moderate | 15 (16.7%) | 25 (27.8%) |  |
| Poor | 13 (14.4%) | 37 (41.1%) |  |
| Tumor size |  |  | 0.162 |
| ≤5cm | 18 (20%) | 30 (33.3%) |  |
| ＞5cm | 10 (11.1%) | 32 (35.6%) |  |
| Vascular and nerve invasion |  |  | 0.147 |
| 0 | 16 (17.8%) | 45 (50%) |  |
| 1 | 12 (13.3%) | 17 (18.9%) |  |

**Supplementary Table 5.** Univariate and multivariate Cox proportional hazards analyses for OS

| Characteristics | Total(N) | Univariate analysis | |  | Multivariate analysis | |
| --- | --- | --- | --- | --- | --- | --- |
|  |  | Odds Ratio (95% CI) | p value |  | Odds Ratio (95% CI) | p value |
| Gender | 90 |  |  |  |  |  |
| 0 | 37 | Reference |  |  |  |  |
| 1 | 53 | 0.522 (-0.386 - 1.430) | 0.161 |  |  |  |
| Age | 90 |  |  |  |  |  |
| ≤ 65 | 55 | Reference |  |  |  |  |
| >65 | 35 | 0.730 (-0.150 - 1.610) | 0.483 |  |  |  |
| T.stage | 90 |  |  |  |  |  |
| 1 | 3 | Reference |  |  | Reference |  |
| 2 | 11 | 0.750 (-1.993 - 3.493) | 0.837 |  | 0.009 (-4.582 - 4.601) | **0.046*** |
| 3 | 53 | 3.579 (1.114 - 6.044) | 0.311 |  | 0.165 (-2.801 - 3.131) | 0.234 |
| 4 | 23 | 13.333 (10.644 - 16.023) | 0.059 |  | 0.332 (-2.928 - 3.592) | 0.507 |
| N.stage | 90 |  |  |  |  |  |
| 0 | 23 | Reference |  |  | Reference |  |
| 1 | 12 | 2.375 (0.762 - 3.988) | 0.293 |  | 1.070 (-0.868 - 3.008) | 0.945 |
| 2 | 18 | 16.625 (15.077 - 18.173) | **< 0.001***** |  | 9.208 (7.230 - 11.185) | **0.028*** |
| 3 | 37 | 171.000 (168.739 - 173.261) | **< 0.001***** |  | 141.627 (139.131 - 144.124) | **< 0.001***** |
| Pathologic differentiation | 90 |  |  |  |  |  |
| Well/Moderate | 40 | Reference |  |  | Reference |  |
| Poor | 50 | 2.575 (1.689 - 3.461) | **0.036*** |  | 3.913 (2.294 - 5.532) | 0.099 |
| Tumor size | 90 |  |  |  |  |  |
| ≤5cm | 48 | Reference |  |  |  |  |
| ＞5cm | 42 | 1.786 (0.904 - 2.667) | 0.197 |  |  |  |
| Vascular and nerve invasion | 90 |  |  |  |  |  |
| 0 | 61 | Reference |  |  | Reference |  |
| 1 | 29 | 7.854 (6.558 - 9.151) | **0.002**** |  | 14.551 (12.435 - 16.667) | **0.013*** |
| INHBB expression | 90 |  |  |  |  |  |
| Low expression | 28 | Reference |  |  | Reference |  |
| High expression | 62 | 1.961 (1.043 - 2.880) | 0.150 |  | 2.383 (0.272 - 4.493) | 0.420 |

**Supplementary figures and figure legends**


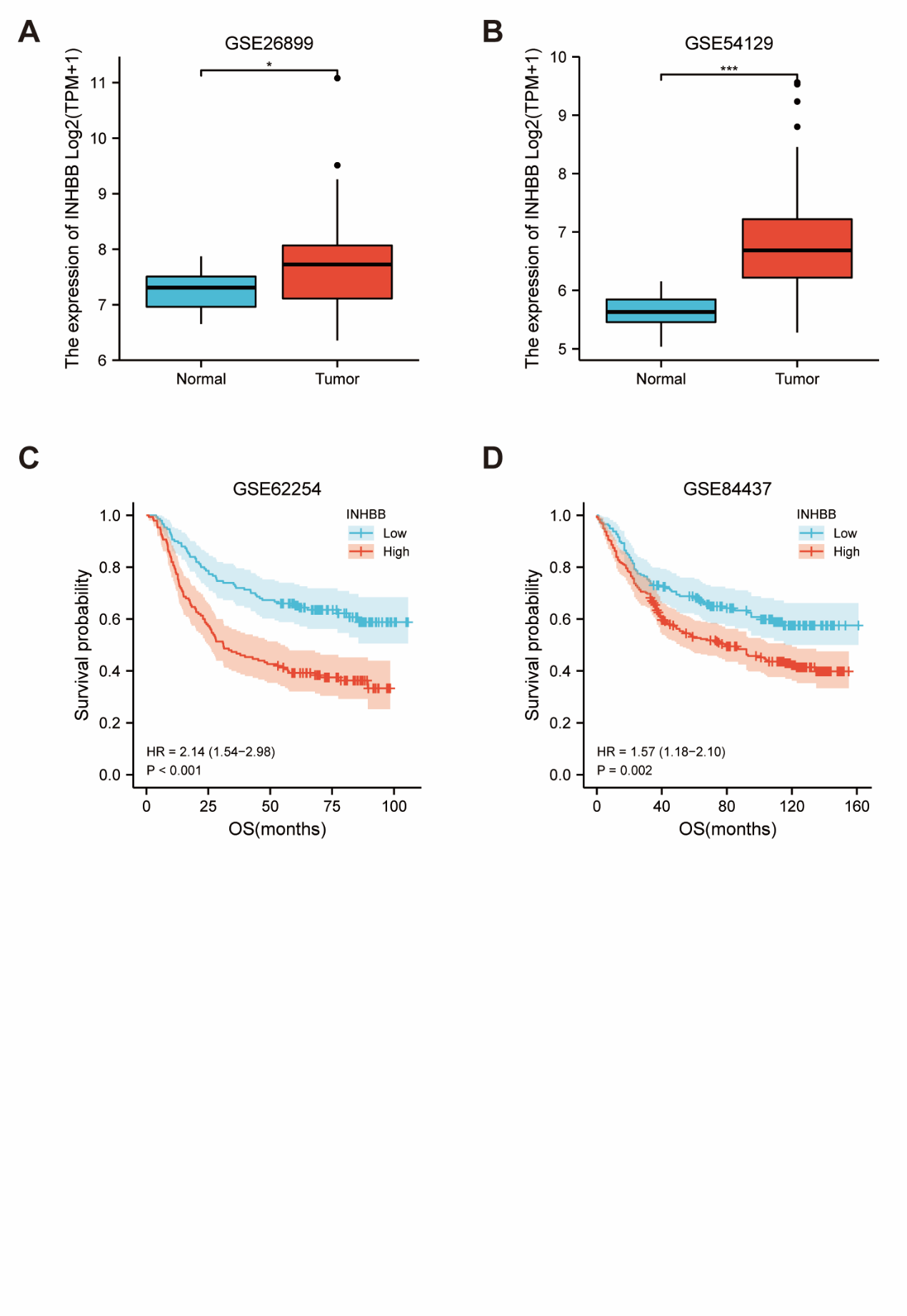


**Figure. S1 The high expression of INHBB identified in GC is correlated with poor prognosis.** A-B. INHBB expression in GC and normal stomach tissues from two GEO databases (A: GSE26899; B: GSE54129). C-D. Overall survival Kaplan-Meier analysis of patients from two GEO databases (C: GSE62254; D: GSE84437). *, P < 0.05; ***, P < 0.001.


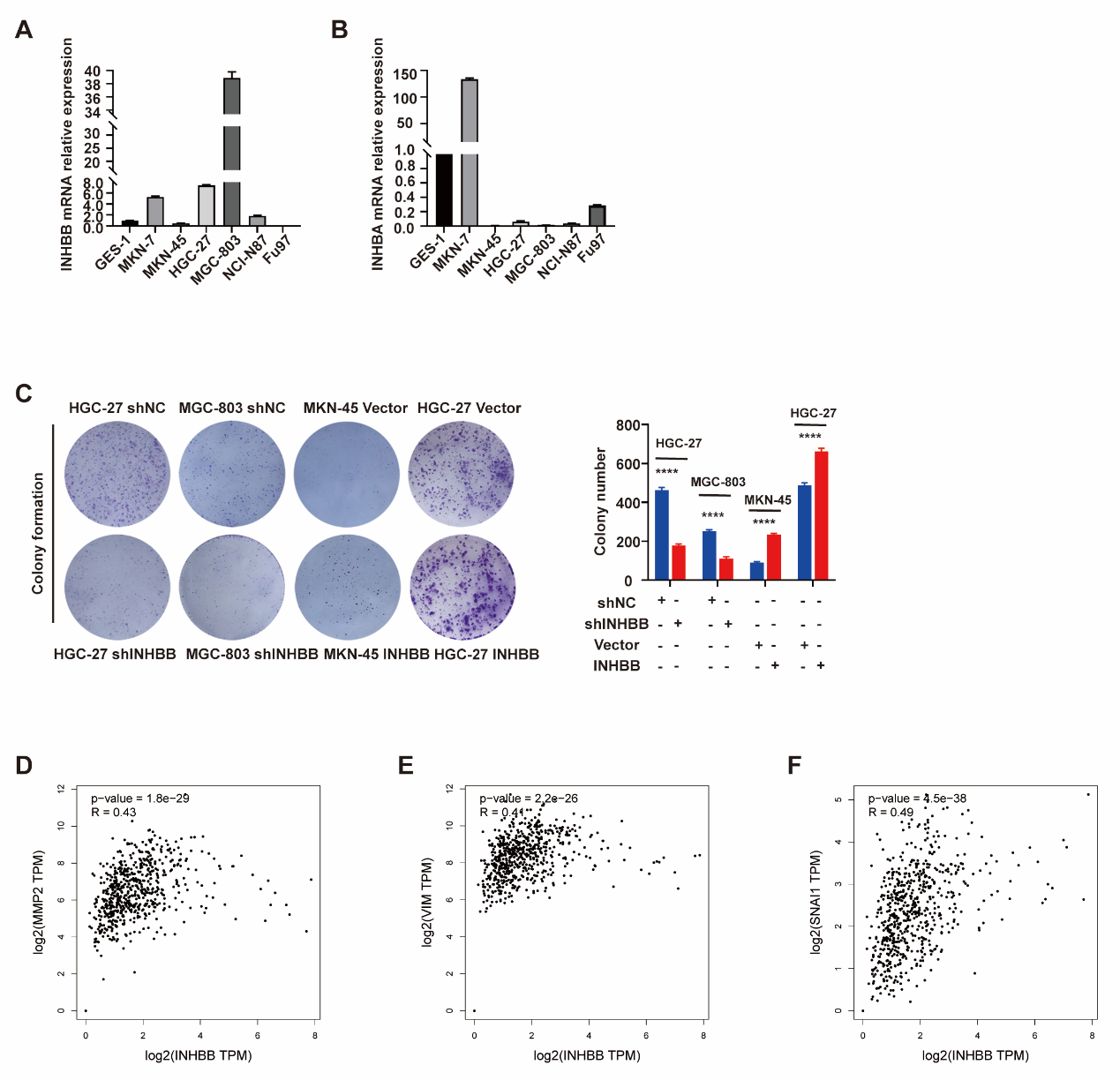


**Figure. S2 INHBB stimulates GC cell proliferation, migration, and invasion *in vitro*.** A-B. mRNA levels of INHBB and INHBA were examined by qRT-PCR in GES-1, MKN-7, MKN-45, HGC-27, MGC-803, NCI-N87 and Fu97 cells. C. Cell proliferation was verified by colony formation assay in HGC-27, MGC-803 and MKN-45 infected with shNC, shINHBB, Vector or INHBB-amplified lentivirus (n=3). D-F. Correlation analysis of mRNA levels between INHBB and MMP2, VIM and SNAI1 based on the TCGA database using GEPIA. ****, P < 0.0001.


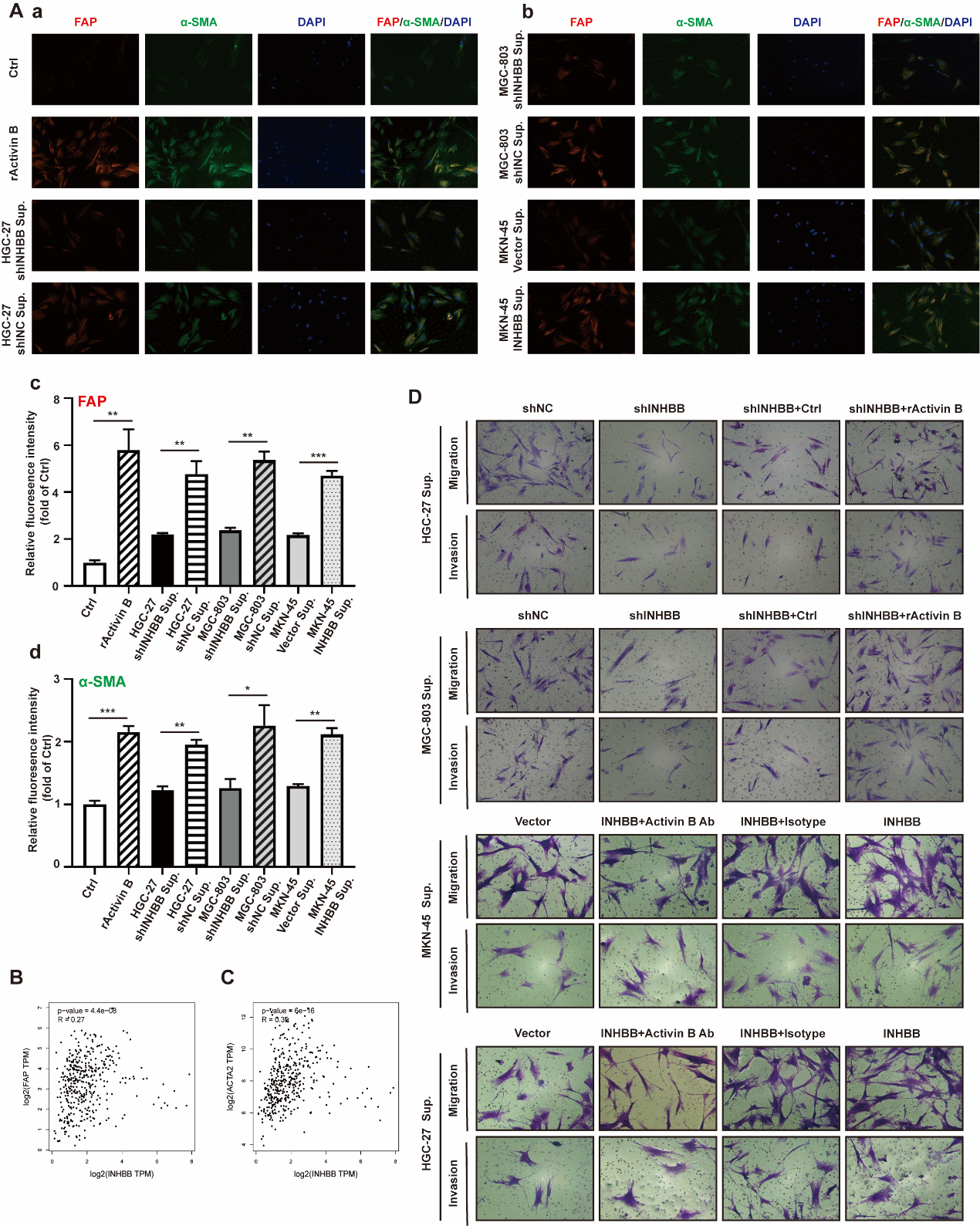


**Figure. S3 GC cell-derived activin B induces a CAF phenotype in fibroblasts.** Aa-d. Effects of activin B on the normal fibroblast’s protein expression levels of α-SMA and FAP as assessed by immunofluorescence assays. Image J was used to measure the area, integrated density and mean integrated density values of two indicators by selecting three fields of view in each picture. Statistical analysis (t-tests) was performed using GraphPad Prism. B-C. Correlation analysis of mRNA levels between INHBB and FAP and ACTA2 based on TCGA database using GEPIA. D. Cell migration and invasion were detected by transwell assay in normal fibroblasts co-cultured with different INHBB-expressing GC cells treated with activin B-neutralizing antibody or exogenous activin B. rActivin B: Recombinant Human Activin B; Sup.: supernatant; Activin B Ab: activin B neutralizing antibody; Isotype: isotype antibody. *, P < 0.05; **, P < 0.01; ***, P < 0.001.


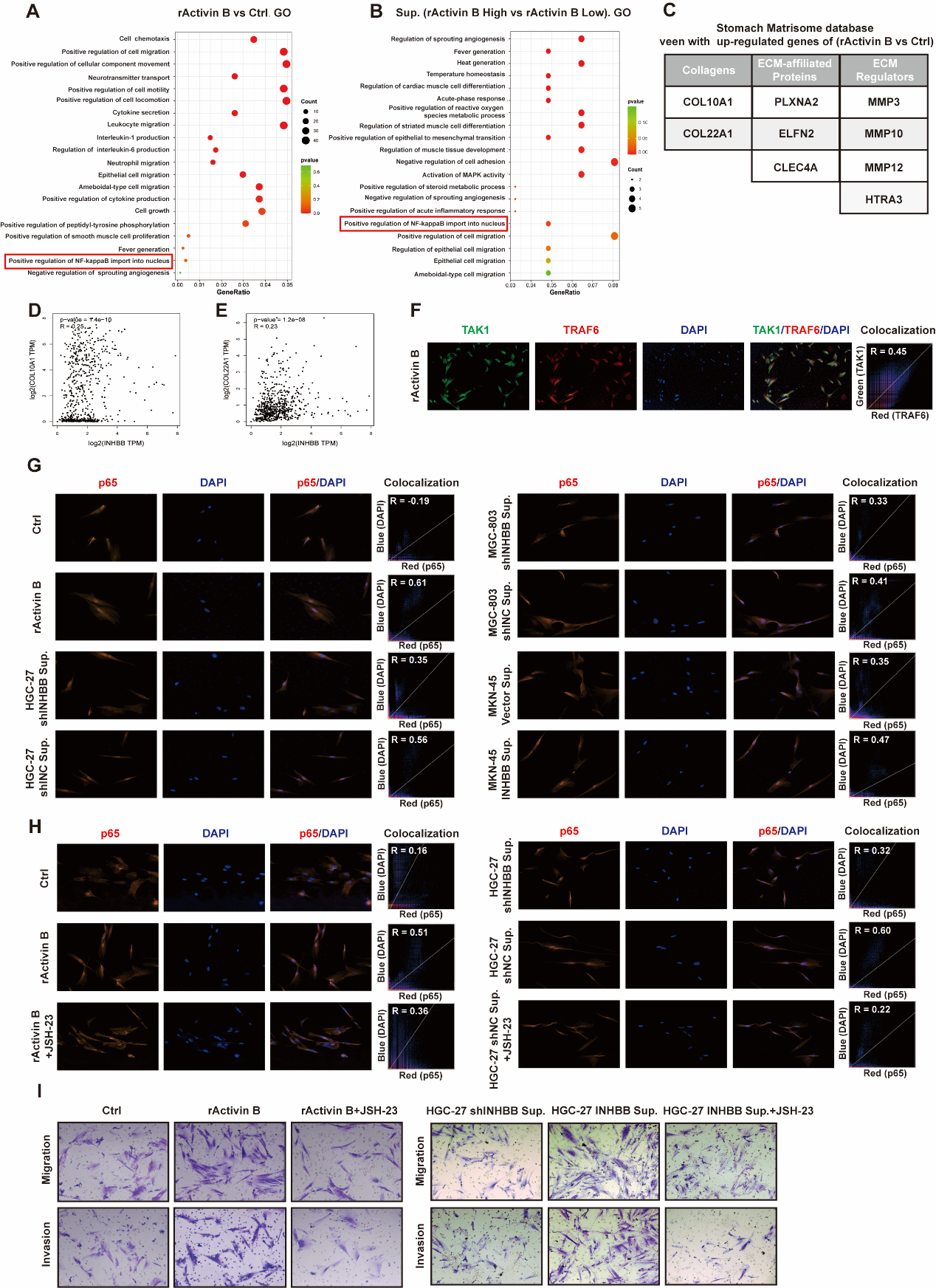


**Figure. S4 GC cell-derived activin B regulates NF-κB activity of fibroblasts through ALK7/TRAF6/TAK1.** A-B. GO pathway enrichment analysis of significantly DEGs. C. This filter showed selected top collagens, ECM-affiliated proteins and ECM regulators that are up-regulated according to the Matrisome database. D-E. Correlation analysis of mRNA levels between INHBB and COL10A1 and COL22A1 based on TCGA database using GEPIA. F. The protein colocalization of TRAF6 and TAK1 in fibroblasts was detected by immunofluorescence assays. Image J plugin (Colocalization-Coloc 2) was used to measure Pearson’s correlation coefficient (R) of two colocalization indicators. G. In fibroblasts co-cultured with activin B or different INHBB-expressing GC cells, nuclear translocation of p65 is examined by immunofluorescence. Image J plugin was used to measure Pearson’s correlation coefficient of two colocalization indicators. H. Immunofluorescence assay evaluating the effect of JSH-23 to the nuclear translocation of p65 in fibroblasts. Image J plugin was used to measure Pearson’s correlation coefficient of two colocalization indicators. I. Fibroblasts under high activin B environment were treated with JSH-23. Transwell assay was used to evaluate cell migration and invasion (n=3). rActivin B: Recombinant Human Activin B; Sup.: supernatant.


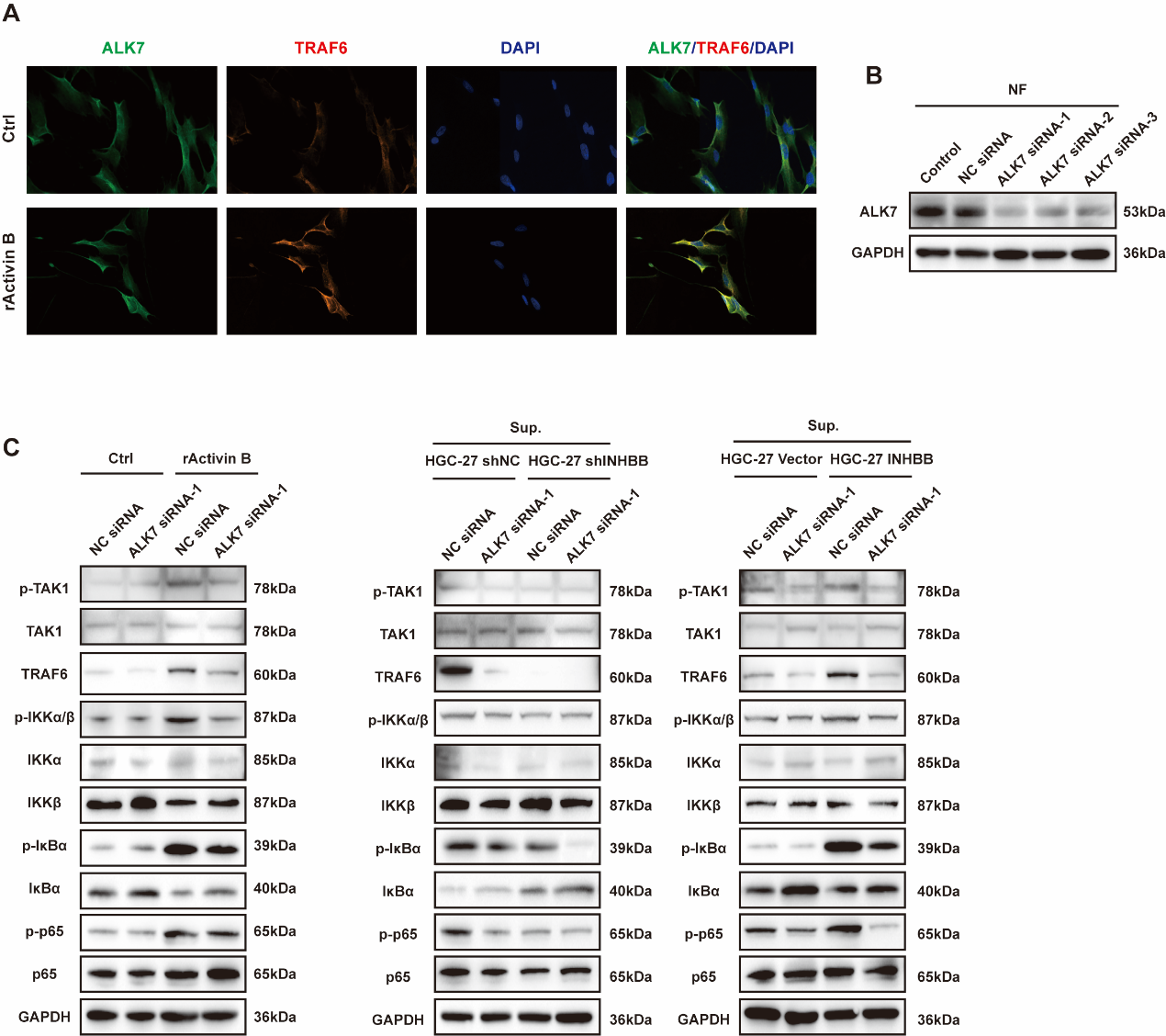


**Figure. S5 GC cell-derived activin B regulates NF-κB activity of fibroblasts through ALK7/TRAF6/TAK1.** A. The protein colocalization of ALK7 and TRAF6 in fibroblasts was detected by immunofluorescence assays. B. ALK7 expression knockdown by siRNA in normal fibroblasts detected by Western blot. C. Western blot analyses of the levels of TRAF6, p-TAK1, p-IKKα/β, p-IκBα, p-p65, total TAK1, IKKα/β, IκBα and p65 in fibroblast co-cultured with activin B or different INHBB-expressing GC cells after transfected with or without ALK7 siRNA. rActivin B: Recombinant Human Activin B; Sup.: supernatant.


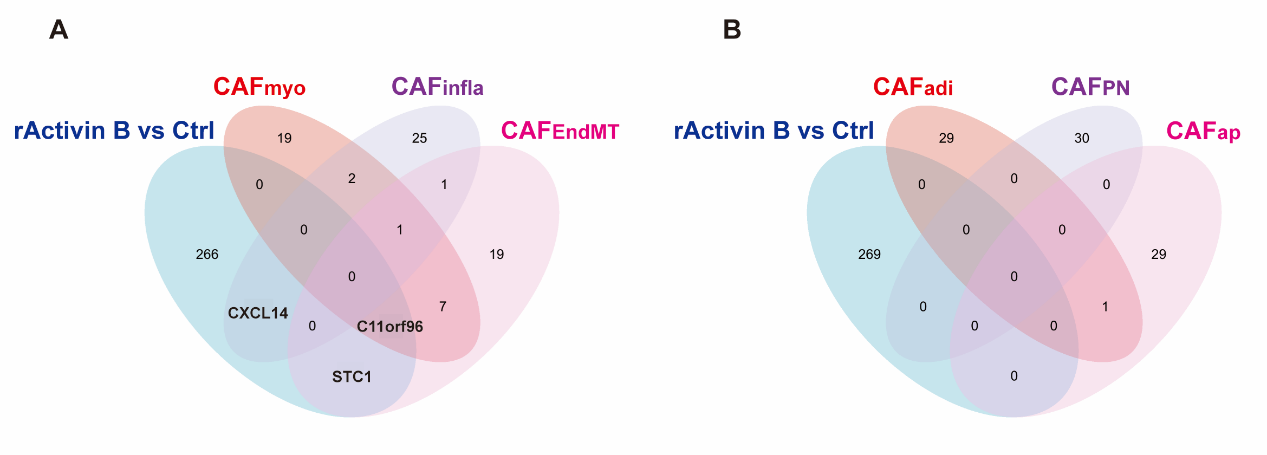


**Figure. S6 Classification of fibroblasts stimulated by activin B.** A-B. Fibroblasts classification Venn plots performed by top molecular markers. rActivin B: Recombinant Human Activin B.


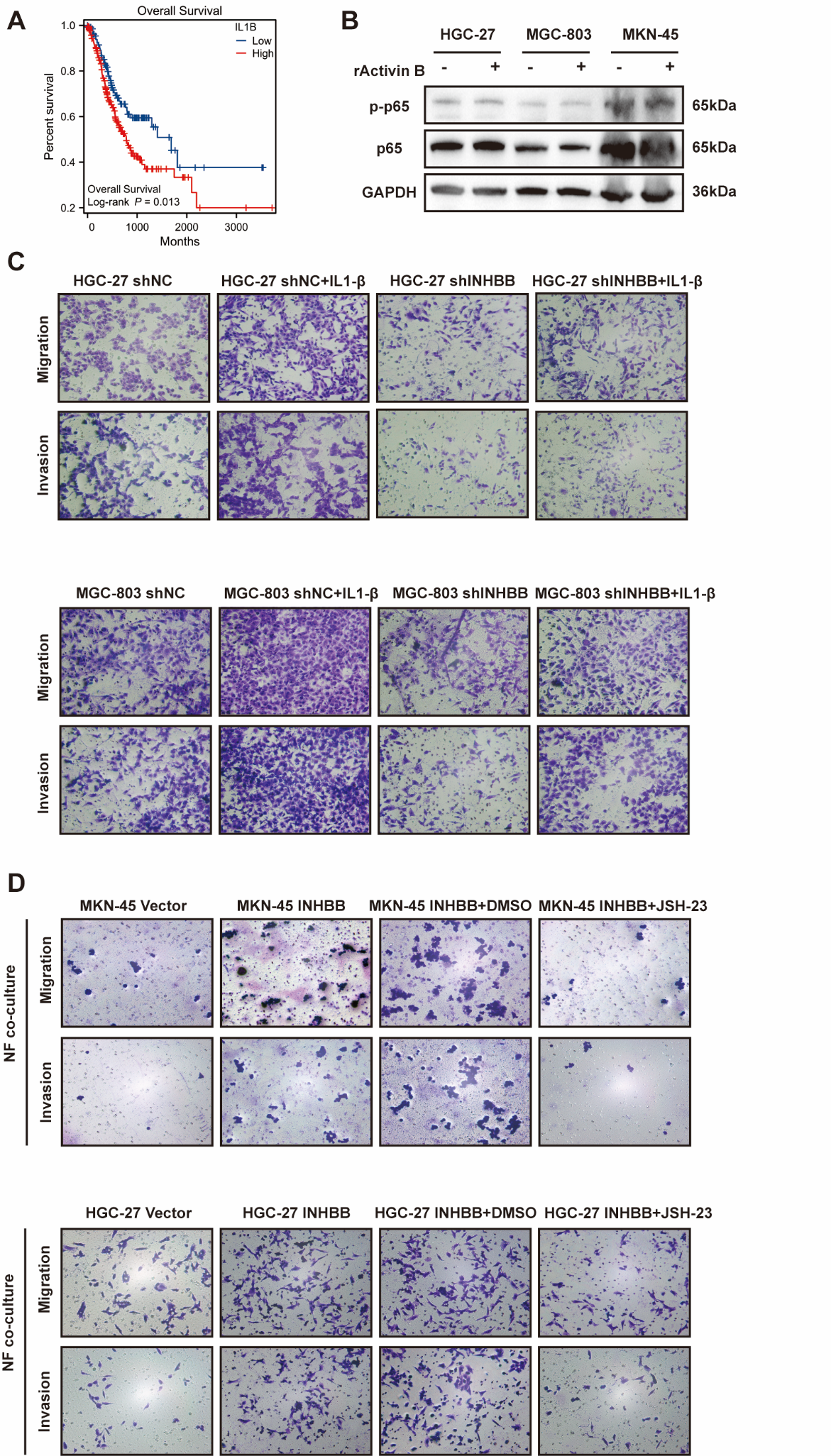


**Figure. S7 Activated fibroblasts regulate GC cell function via the** **IL-1β/p65 pathway.** A. Overall survival analysis of the GC cohort based on TCGA database using GEPIA. B. Western blot analyses of the levels of p-p65 and p-65 in GC cells treated and untreated with activin B. C. Cell migration and invasion were detected by transwell assay in GC cells with or without IL-1β co-culture (n=3). D. Cell migration and invasion were detected by transwell assay in GC cells in fibroblasts co-cultured system with or without JSH-23 co-culture (n=3). rActivin B: Recombinant Human Activin B.


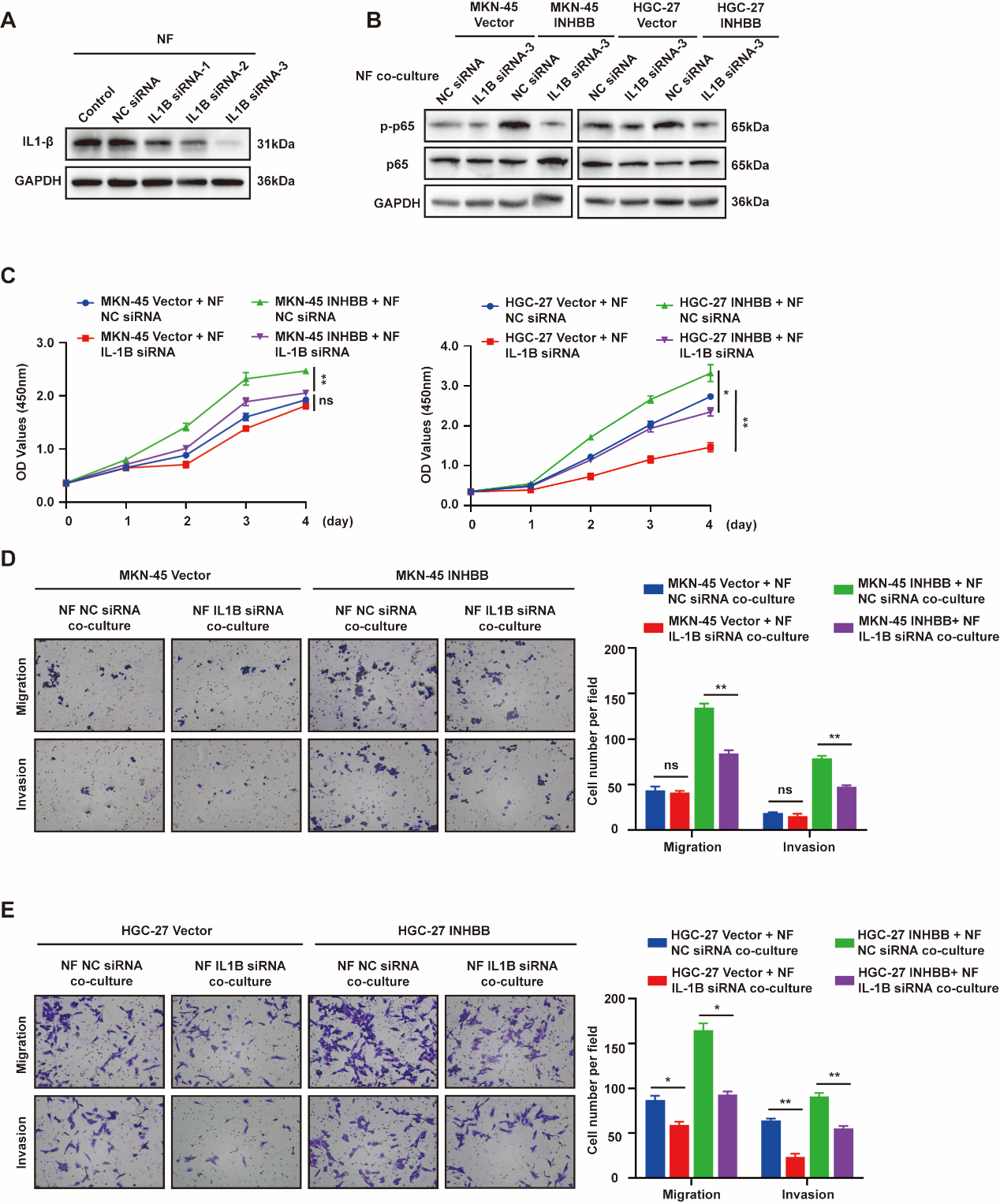


**Figure. S8 Activated fibroblasts regulate GC cell function via the IL-1β/p65 pathway.** A. IL-1β expression knockdown by siRNA in normal fibroblasts detected by Western blot. B. Western blot analyses of the levels of p-p65 and p-65 in GC cells co-cultured with normal fibroblasts which were transfected with or without IL-1β siRNA. C. Cell proliferation was verified by CCK-8 assay. The value of the absorbance (at 450 nm) was recorded from 0 h to 96 h in GC cells co-cultured with normal fibroblasts which were transfected with or without IL-1β siRNA (n=3). D-E. Cell migration and invasion were detected by transwell assay in GC cells co-cultured with normal fibroblasts which were transfected with or without IL-1β siRNA (n=3). *, P < 0.05; **, P < 0.01.


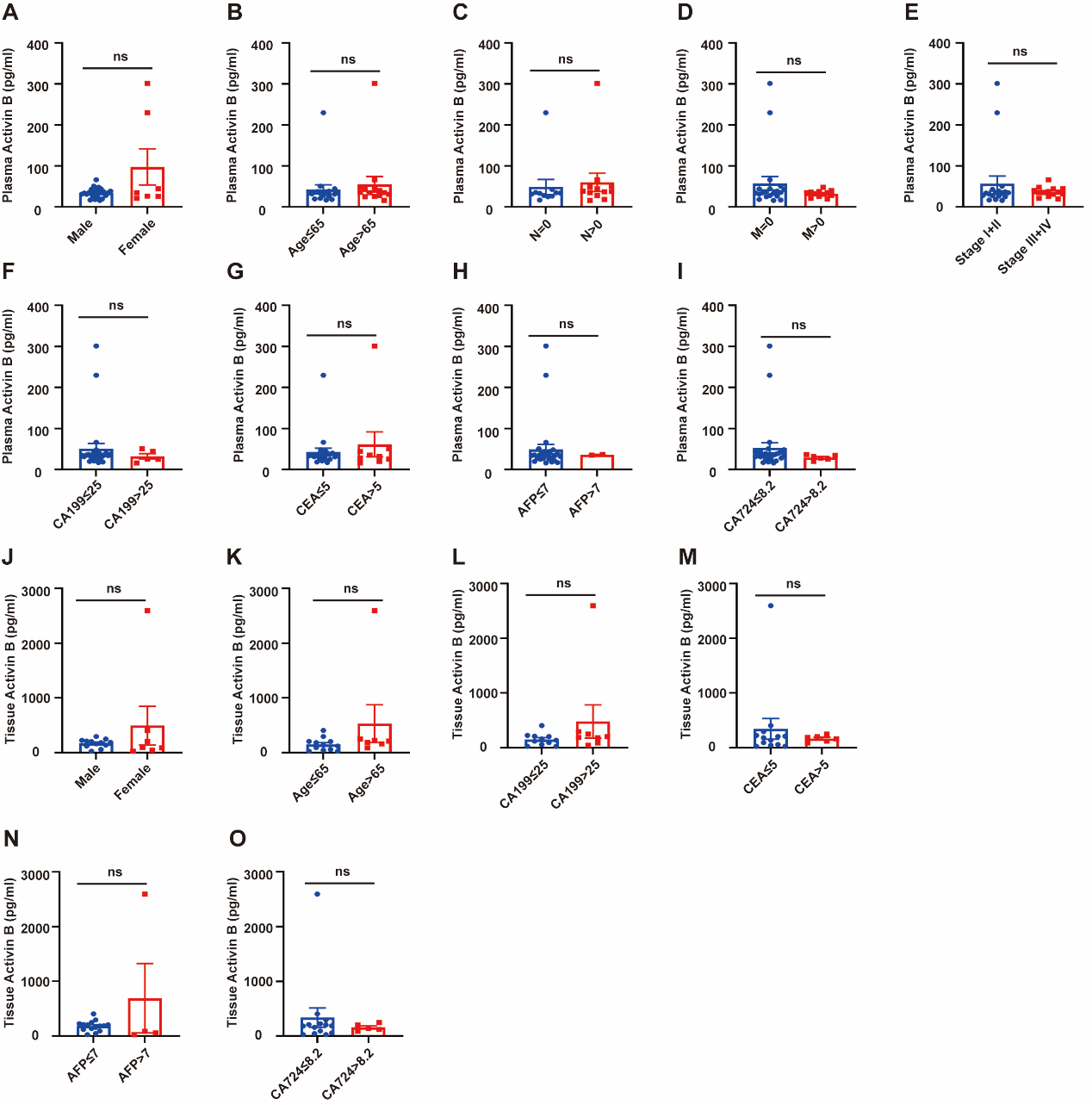


**Figure. S9 Elevated circulating and tissue activin B associates with GC.** A-I. Boxplots representing the plasma activin B level in the different clinicopathological features of GC patients (n=32). J-O. Boxplots representing the tissue activin B level in the different clinicopathological features of GC patients (n=19).


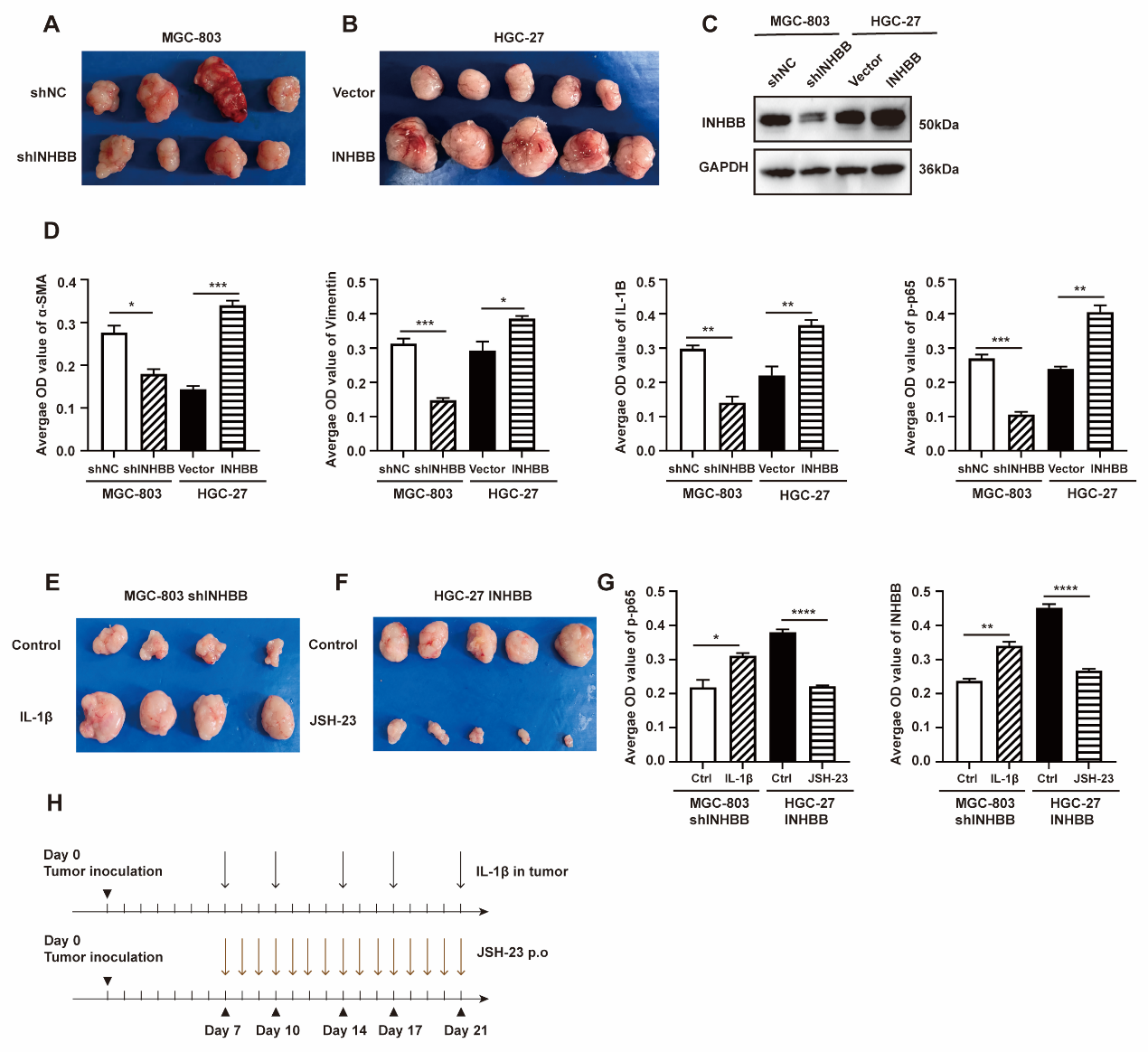


**Figure. S10 INHBB promotes GC progression *in vivo*.** A-B. Tumor tissues from mice bearing xenografts. C. The levels of INHBB in tissues harvested from xenograft formed from MGC-803 or HGC-27. D. The statistical analyses of collagen, α-SMA, Vimentin, IL-1β and p-p65 expression were evaluated by IHC in tissues of xenograft. All results were analyzed with statistical analysis using Image J and GraphPad Prism. Image J was used to measure the area, optical density (OD) and mean optical density values of IHC by selecting three fields of view in each section. E-F. Tumor tissues from mice bearing xenografts. G. The statistical analyses of p-p65 and INHBB expression were evaluated by IHC in tissues of xenograft. All results were analyzed with statistical analysis using Image J and GraphPad Prism. Image J was used to measure the area, optical density (OD) and mean optical density values of IHC by selecting three fields of view in each section. H. GC cells and fibroblasts were inoculated subcutaneously in nude mice. Follow-up treatment (JSH-23, IL-1β or control reagent) was then performed as shown. *, P < 0.05; **, P < 0.01; ***, P < 0.001; ****, P < 0.0001.
