# Supplementary material for: Paracrine activin B-NF-κB signaling shapes an inflammatory tumor microenvironment in gastric cancer via fibroblast reprogramming
Source: J Exp Clin Cancer Res. 2023 Oct 19;42:269. doi: 10.1186/s13046-023-02861-4 (PMC10585924; doi:10.1186/s13046-023-02861-4)
Supplement: Supplementary file 1 — Supplementary Material 1 [file 13046_2023_2861_MOESM1_ESM.docx]

**Response Letter – Manuscript JECC-D-23-01338**

We feel great thanks for your professional review work on our article. As you concerned, there are two problems needed to be addressed. According to your great suggestions, we have made some corrections to our manuscript, details are listed below.

(Q=Question; A=Answer)

**Reviewers’ Comments:**

**Reviewer #2 (Remarks to the Author):**

I agree the author's revise in large. There are some issues that need to be addressed.

**A: We sincerely appreciate for your valuable comments on our manuscript.**

**Q1:** What is the receptor of activin B which expressed on fibroblast membrane, and how conduct the signaling to TAK1/NF-κB?

**A1: We do thank the reviewer for this reasonable concern. As reported, ALK7 is the principal type I receptor of activin B and is the main receptor for downstream signaling^1,2^.**

**To further validate the molecular mechanism by which activin B-ALK7 plays a role in the activation of normal fibroblasts of GC, firstly, we detected ALK7 and TRAF6 colocalization in fibroblasts by immunofluorescence assay (Fig. 1A). ALK7 is known to be a transmembrane protein and TRAF6 is known to be a cytoplasmic protein. In fibroblasts stimulated with activin B, compared with the control group, TRAF6 was visualized the local aggregation on the cell membrane, which was consistent with our previous conjecture. We considered transmembrane receptor ALK7 could bind TRAF6 interaction motifs to activate TRAF6 by promoting oligomerization. Additionally, we established normal gastric fibroblasts with ALK7 silence through siRNA (Fig. 1B). In activin B stimulation system with or without transfect with ALK7 siRNA, we detected TRAF6 and TAK1 phosphorylation of fibroblasts by Western blot. The results found that the up-regulation of TRAF6 and phosphorylation of TAK1 activated by activin B was inhibited in ALK7 silent fibroblasts. Furthermore, we evaluated the phosphorylation of Iκ Bα, IKK α/β and p65 in fibroblasts stimulated by activin B with or without transfect with ALK7 siRNA. The results showed that ALK7 silence could reverse the up-regulation of phosphorylation of IκBα, IKK α/β and p65 in fibroblasts induced by activin B. The above results also held in GC cells co-cultured system with or without transfect with ALK7 siRNA (Fig. 1C). Overall, these results indicated that activin B activates and conducts the signaling to fibroblasts through ALK7-TRAF6-TAK1-NF-κB axis.**

**Figure 1. Gastric cancer (GC) cell-derived activin B regulates NF-κB activity of fibroblasts through ALK7/TRAF6/TAK1.** A. The protein colocalization of ALK7 and TRAF6 in fibroblasts was detected by immunofluorescence assay. B. ALK7 expression knockdown by siRNA in normal fibroblasts was verified by Western blot. C. Western blot analyses of the levels of p-TAK1, TAK1, TRAF6, p-IKKα/β, IKKα, IKKβ, p-IκBα, IκBα, p-p65 and p65 in fibroblast co-cultured with activin B or different INHBB-expressing GC cells after transfected with or without ALK7 siRNA. rActivin B: Recombinant Human Activin B; Sup.: supernatant.

**Q2:** The novelty of this manuscript is limited.

**A2: We do thank the reviewer for this thoughtful concern. We also regret about some novelty limitations in this study, such as large studies among tumor microenvironment (TME), inflammation and tumorigenesis, many connections between fibroblasts and their effectors in TME. Even so, comparing with previous studies, our work also owns the following shining points.**

1. **For the first time, we proposed a positive correlation between the concentration of plasma tumor biomarker CA125 and the concentration of activin B in GC patients.**
2. **In our study, we established a positive regulatory feedback loop between GC cells and fibroblasts. Activin B, the dimer structure formed by INHBB subunit, activates NF-κB pathway by controlling the autoubiquitination of TRAF6 and inducing TAK1 phosphorylation in fibroblasts. Activation of NF-κB releases IL-1β, which facilitates the phosphorylation of p65 and increases GC cells proliferation and invasion. Furthermore, p65 directly promotes INHBB transcription and induces INHBB expression. Altogether, our finding identified, for the first time, that activin B is a key mediator of GC progression both in vitro and in vivo.**
3. **Our research revealed that INHBB creates a local tumor-promoting inflammatory environment between GC cells and fibroblasts. The inflammatory TME provided by activin B in GC provides a new perspective for gastric cancer TGF-β inhibition.**

**In conclusion, it is precisely because the crucial roles of fibroblasts crosstalk in GC initiation and progression, our findings have important implications for facilitating the translation of fibroblasts-based therapeutic targets into clinical trials.**

**References:**

1	Lodberg, A. Principles of the activin receptor signaling pathway and its inhibition. *Cytokine Growth Factor Rev* **60**, 1-17, doi:10.1016/j.cytogfr.2021.04.001 (2021).

2	Bernard, D. J., Lee, K. B. & Santos, M. M. Activin B can signal through both ALK4 and ALK7 in gonadotrope cells. *Reprod Biol Endocrinol* **4**, 52, doi:10.1186/1477-7827-4-52 (2006).
